# A TALEN-Exon Skipping Design for a Bethlem Myopathy Model in Zebrafish

**DOI:** 10.1371/journal.pone.0133986

**Published:** 2015-07-29

**Authors:** Zlatko Radev, Jean-Michel Hermel, Yannick Elipot, Sandrine Bretaud, Sylvain Arnould, Philippe Duchateau, Florence Ruggiero, Jean-Stéphane Joly, Frédéric Sohm

**Affiliations:** 1 UMS 1374, AMAGEN, INRA, Jouy en Josas, Domaine de Vilvert, France; 2 UMS 3504, AMAGEN, CNRS, Gif-sur-Yvette, France; 3 UMR 9197, INRA-CASBAH team, NEURO-Psi, CNRS, Gif sur Yvette, France; 4 UMR 9197, DECA team, NEURO-Psi, CNRS, Gif sur Yvette, France; 5 UMR 5242, Institut de Génomique Fonctionnelle de Lyon, ENS de Lyon, CNRS, Université Lyon 1, Lyon, France; 6 CELLECTIS SA, Paris, France; Texas A&M University, UNITED STATES

## Abstract

Presently, human collagen VI-related diseases such as Ullrich congenital muscular dystrophy (UCMD) and Bethlem myopathy (BM) remain incurable, emphasizing the need to unravel their etiology and improve their treatments. In UCMD, symptom onset occurs early, and both diseases aggravate with ageing. In zebrafish fry, morpholinos reproduced early UCMD and BM symptoms but did not allow to study the late phenotype. Here, we produced the first zebrafish line with the human mutation frequently found in collagen VI-related disorders such as UCMD and BM. We used a transcription activator-like effector nuclease (TALEN) to design the *col6a1^ama605003^*-line with a mutation within an essential splice donor site, in intron 14 of the *col6a1* gene, which provoke an in-frame skipping of exon 14 in the processed mRNA. This mutation at a splice donor site is the first example of a template-independent modification of splicing induced in zebrafish using a targetable nuclease. This technique is readily expandable to other organisms and can be instrumental in other disease studies. Histological and ultrastructural analyzes of homozygous and heterozygous mutant fry and 3 months post-fertilization (mpf) fish revealed co-dominantly inherited abnormal myofibers with disorganized myofibrils, enlarged sarcoplasmic reticulum, altered mitochondria and misaligned sarcomeres. Locomotion analyzes showed hypoxia-response behavior in 9 mpf *col6a1* mutant unseen in 3 mpf fish. These symptoms worsened with ageing as described in patients with collagen VI deficiency. Thus, the *col6a1^ama605003^*-line is the first adult zebrafish model of collagen VI-related diseases; it will be instrumental both for basic research and drug discovery assays focusing on this type of disorders.

## Introduction

Collagen type VI proteins are found in the extracellular matrix; they are associated with interstitial type I–III collagen fibers, in connective tissues of skin [1] and skeletal muscle [2–4]. Collagen VI is involved in cell-cell attachment and interact with collagen IV of the basement membrane [5], thus playing an important role in muscle maintenance. The predominant form of collagen VI is a heterotrimer made of three polypeptide chains: alpha 1, alpha 2 and alpha 3 that are encoded by the *COL6A1*, *COL6A2* and *COL6A3* genes respectively. Each alpha chain contains a short triple helical collagenous domain, flanked by N- and C-terminal globular domains of variable size [6]. In the functional collagen VI, the three alpha chains combine intracellularly into triple helical monomer that assembles into antiparallel dimer, and then two dimers form a tetramer. The tetramers are secreted into the extracellular matrix where they assemble into beaded microfibrils, the functional unit of collagen VI [7–10]. In muscle, interstitial fibroblasts are the main source of collagen VI which is secreted in the endomysium surrounding the muscle fibers [3].

In humans, the deficiency of *COL6* genes expression is associated with numerous diseases [4,11–15]. Mutations in one of the three genes have been demonstrated to cause the onset of muscular dystrophy [4,16,17,12,13]. Collagen VI-related disorders account for 7 to 19% of congenital muscular dystrophies as reported in several studies on cohorts of patients of different ethnicities [18–20]. Bethlem myopathy (BM, MIM #158810) was first described in 1976 as a benign myopathy with a childhood onset and a slow progression into adulthood [21]. BM represents the mildest form of the phenotypic spectrum of collagen VI-related disorders and Ullrich congenital muscular dystrophy (UCMD, MIM #254090) is at the most severe end of the spectrum [4,13,14]. UCMD is characterized by clinico-pathological hallmarks such as kyphoscoliosis, torticollis, follicular hyperkeratosis, excessive scar formation following skin trauma, distal joint laxity, proximal joint contractures, protruding calcanei, scoliosis and early respiratory insufficiency [1,22,23]. BM and UCMD are rare diseases with reported incidences in Northern England below 1:100,000 and 1:700,000 respectively [24]. Muscle biopsies of patients with BM present characteristics of a myopathic rather than dystrophic histology i.e. muscle fibers exhibit abnormally variable sizes, with an increase in the number of internal nuclei while typically no fiber necrosis is found [2,25]. In UCMD, respiratory problems have been reported at early disease onset, but this outcome remains infrequent in BM [4,26,27]. UCMD progresses slowly and worsens with ageing. Ultimately more than half of the patients end up with severe impairment of their walking ability [13,28]. Currently, BM and UCMD remain without cure.

BM and UCMD syndromes have been associated with mutations in one of the *COL6A1*, *COL6A2* or *COL6A3* genes [4,13]. Most of these mutations are substitutions that modify a splice site or create a premature stop codon. These mutations frequently disrupt the collagen protein structure by perturbing normal helix folding. In the case of BM, mutations frequently affect the correct assembly of collagen VI fibers or prevent the intracellular assembly and secretion of tetramers in the extracellular matrix, eventually diminishing the amount of functional collagen VI protein [4,13,29].

Mouse and zebrafish models reproducing mutations found in patients have contributed to a better understanding of the collagen VI-related diseases. In mouse lines, each of the three genes encoding collagen VI has been individually inactivated and the corresponding alterations in muscle were extensively studied in these mutants [30–35]. Similarly, zebrafish models with different forms of altered collagen VI chains were obtained by the injection of morpholinos [36,37]. However, the morphants only allowed the study of transient phenotypes in embryos up to 2 days post-fertilization (dpf). These studies have led to the identification of histopathological abnormalities and severe ultrastructural changes in skeletal muscles. In particular, abnormal mitochondria and sarcoplasmic reticulum (SR) were observed as well as defective autophagy [36,38]. In addition, increased levels of apoptosis were detected in skeletal muscles of most animal models except in the *Col6a3* deficient mice [34]. Although potential treatment strategies have been tested on collagen VI-deficient mice as well as on zebrafish with a more severe UCMD-like phenotype, no pharmacological studies have been performed on zebrafish morphants modeling the milder BM-like conditions [36,39] and there were limited to very young fish.

In the present study, we used a transcription activator-like effector nuclease (TALEN [40]) to disrupt an essential splice site resulting in an in-frame skipping of exon 14 in the N-terminal portion of the collagenous domain of the Col6a1 protein chain. We reproduced in zebrafish a co-dominant mutation frequently found in BM patients that prevents the assembly of collagen VI dimers and tetramers and inhibits secretion of tetramers into the extracellular matrix [2,4,11,29]. This mutation enabled us to generate and characterize the first mutant zebrafish line with altered collagen type VI alpha-1 chain.

The resulting *col6a1* mutants (*col6a1* ^*ama605003*^ hereafter and in ZFIN and ZIRC databases) represent the first model to study collagen VI-related disorders based on a stable zebrafish line and will give the opportunity to study the disease’s mechanisms and progression from embryo to ageing fish. Moreover, the *col6a1* ^*ama605003*^ line will facilitate the search for a treatment for these disorders since zebrafish are particularly amenable to pharmacological screens.

## Materials and Methods

### Ethics Statement

Zebrafish of the strain TU were raised and bred according to standard procedures. The creation and maintenance of zebrafish with mutant collagen VI was approved by the local ethics committee. All anesthesia and lethal procedures were performed in tricaine (MS222) solutions as recommended for zebrafish, and all efforts were made to reduce suffering. We confirm that all ethical procedures presented in this study have been approved by the french Comité d’Étique en Expérimentation Animale n°59, Ministère de l’Enseignement Supérieur et de la Recherche (agreement number pending).

All chemicals were purchased from Sigma-Aldrich, unless specified otherwise.

### Generation of TALE nuclease coding sequences and molecular cloning

**Fig 1:**
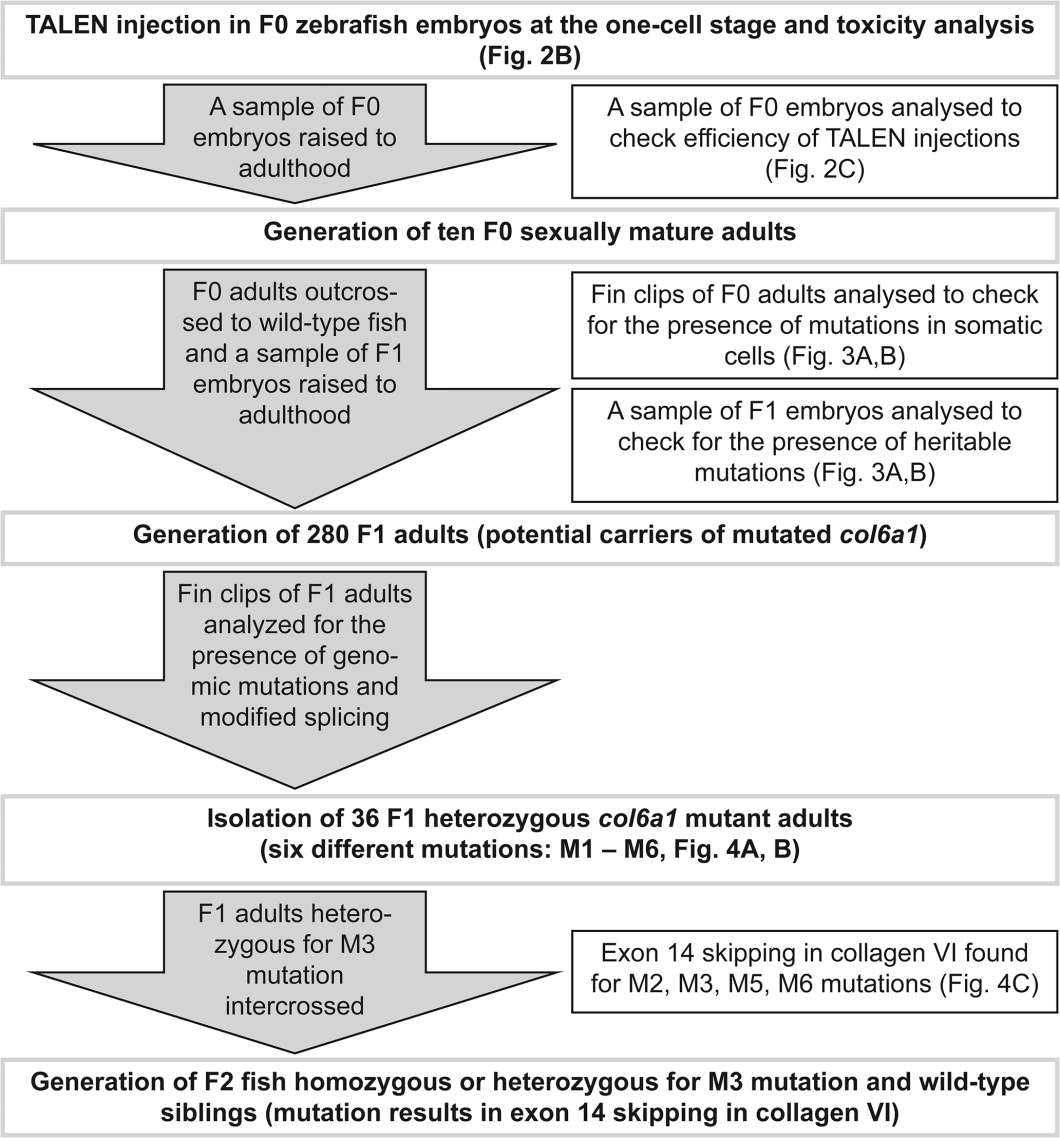
Flowchart for the generation of TALEN-mediated targeted genomic modifications within the *col6a1* gene from the injection at the one-cell stage to the generation of the stable *col6a1* ^*ama605003*^ mutant zebrafish line. The steps performed to establish a zebrafish line with skipping of exon 14 in collagen VI are outlined.

**Fig 2:**
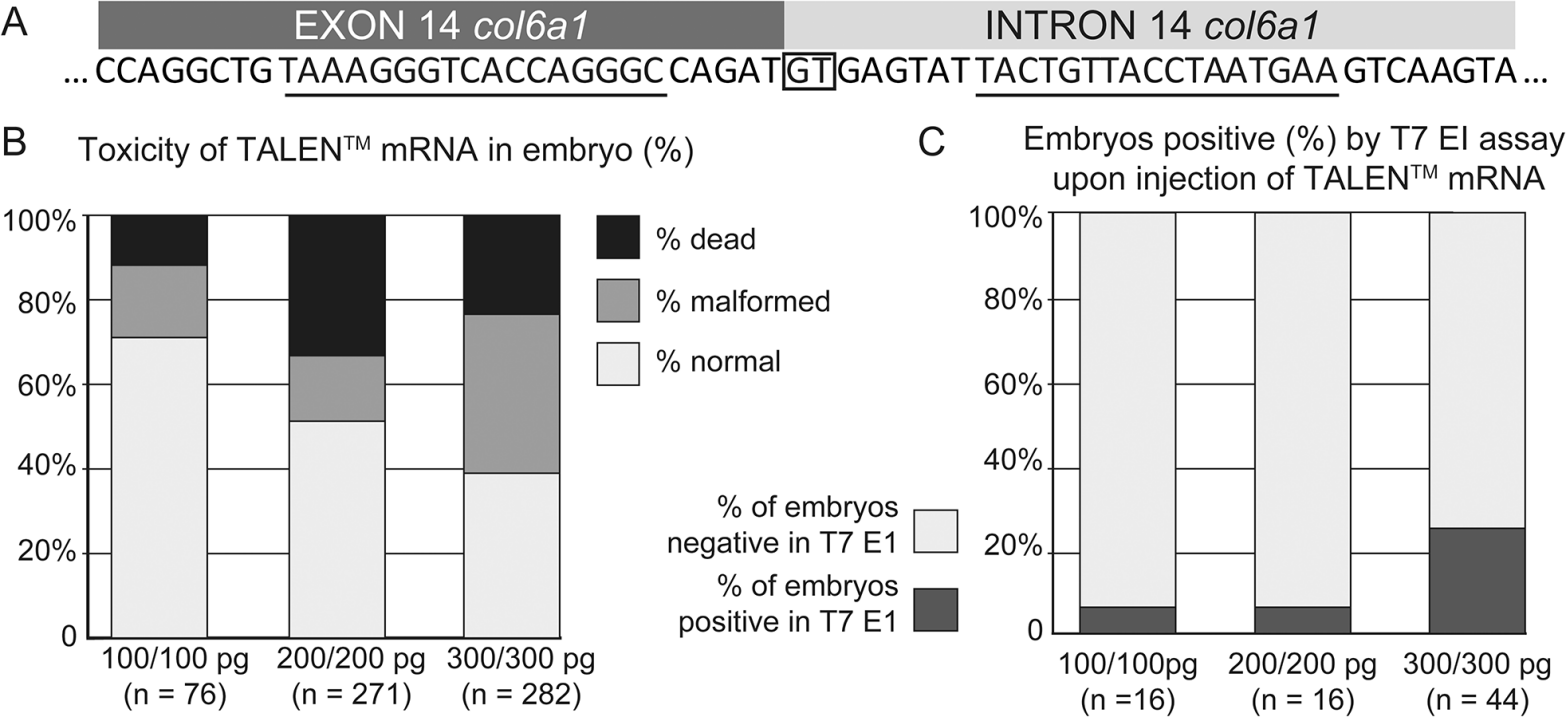
Generation of somatic mutations in the *col6a1* gene in zebrafish. (A) Organization of exon-14 and intron-14 junction and positioning of the TALEN monomer binding sites (underlined). The splice donor site is boxed. (B) Toxicity of the TALEN injection in zebrafish. The proportion of dead, malformed and normal embryos at 2 dpf is shown for each injected concentration. The number of injected embryos is indicated in brackets. (C) T7 EI assay on a representative sample of the injected embryos at 2 dpf, for each condition. The proportion of positive and negative embryos is shown for each injected concentration. The number of injected embryos is indicated in brackets. The relative efficiency of the TALEN doses was not significantly different (p-value 0.1534 exact two-tailed Fisher’s test).

The steps performed to establish a zebrafish line with skipping of exon 14 in collagen VI are outlined in the flowchart (Fig 1). TALE nuclease (TALEN) coding sequences were obtained from CELLECTIS SA bioresearch. The DNA binding sites for the TALEN pair targeting *col6a1* were (5’ to 3’): left site TAAAGGGTCACCAGGGC, right site TTCATTAGGTAACAGTA (Fig 2A). Coding sequences of TALE nucleases were subcloned in a derivative of the pSpe3-RfA vector for mRNA *in vitro* synthesis [41] between the 5’ and 3’ globin UTRs included in the vector. The integrity of the final constructs was verified by sequencing.

### *In vitro* mRNA synthesis

The final TALEN plasmids were linearized by digestion with *KpnI*, the DNAs purified and used as template for *in vitro* mRNA synthesis by the mMESSAGE mMACHINE T3 Transcription Kit (Life Technologies). mRNAs were *in vitro* polyadenylated with the Poly(A) Tailing Kit (Life Technologies) and then purified using the NucleoSpin RNA Clean-up Kit (Macherey-Nagel).

### Injection of zebrafish embryos

Zebrafish embryos at the one-cell stage were injected with three different doses of TALEN mRNAs (200-400-600 pg/pair), using a Picospritzer injector (Science Products). The toxicity of the injected solutions was determined at 24 hpf by calculating the proportion of dead and malformed embryos for each dose.

### Lysis of embryos and fin clips

Normal zebrafish embryos of 2 dpf were euthanized with a lethal dose of tricaine (MS222) buffered with sodium bicarbonate, placed individually in 50 μl of lysis buffer with composition 10 mM Tris-HCl pH 8.0, 50 mM KCl, 0.3% Tween 20, 0.3% Igepal CA-630, 4 mM EDTA, supplemented with proteinase-K to 250 ng/μl just before use. Lysis was performed for 16 h at 55°C and proteinase-K was subsequently inactivated by incubation for 10 min at 95°C. Lysates were stored at -20°C. For fin clip lysates, adult fish (3 mpf) were anesthetized with 0.5 mg/mL tricaine buffered with sodium bicarbonate and biopsies from the caudal fin removed with a sharp blade. Fin clip biopsies were lysed in the same manner as embryos.

### PCR on embryo or fin clip lysates

**Fig 3:**
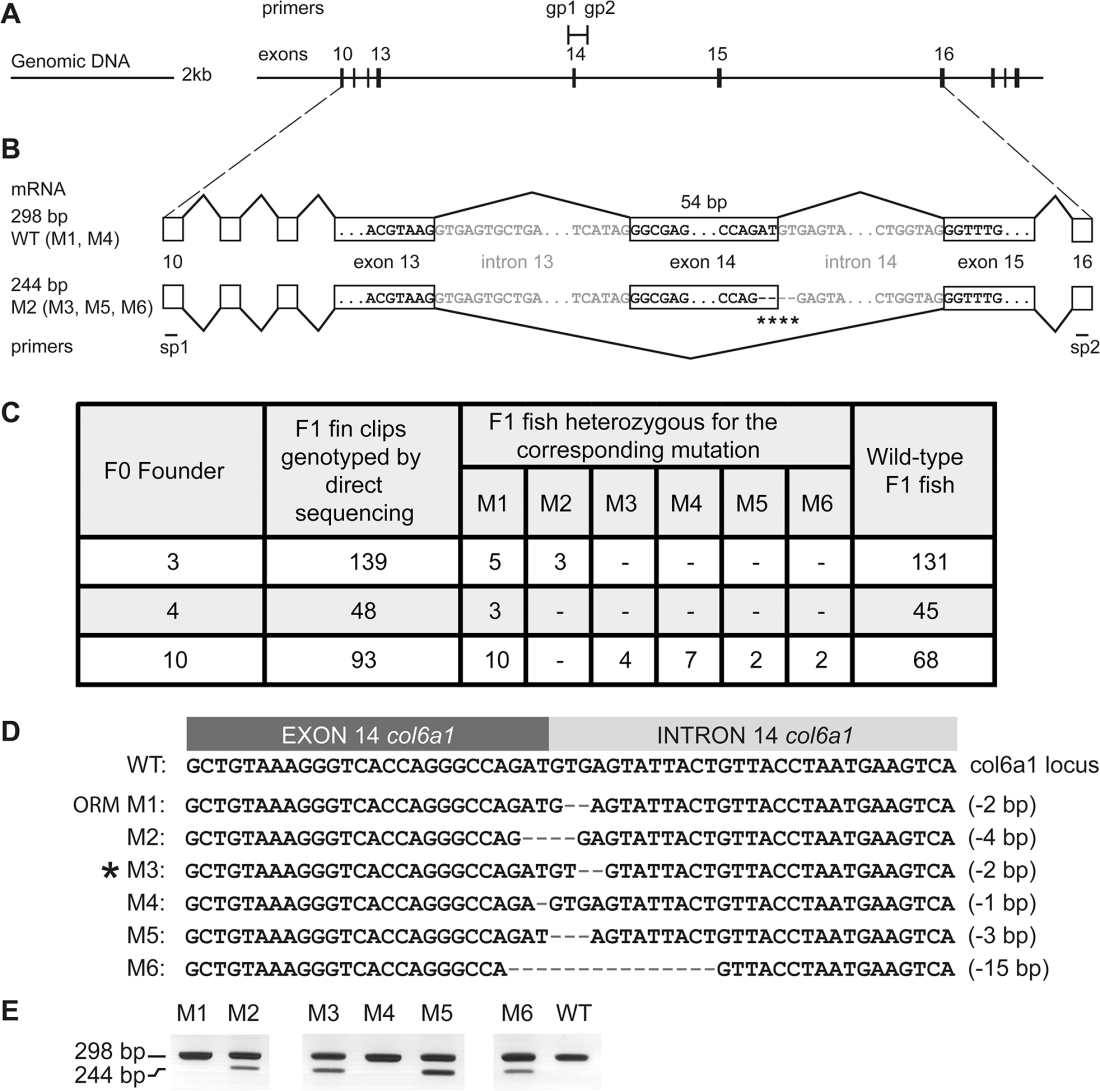
Organization of the targeted locus and validation of the presence of alternatively spliced *col6a1* mRNA in the mutants. (A) Organization of the genomic locus surrounding the col6a1 exon 14. Scale in kb is shown on the left-hand side. Exons 10 to 19 are represented by vertical dashes. Introns are symbolized by a continuous line. Genotyping primers gp1 and gp2 are represented on top. (B) The mRNA structures of wild type (WT, 298 bp) and exon 14-skipped forms (244 bp) are represented. The scheme represents the mRNA with exon skipping of the mutant M2 (deleted bases are symbolized by stars). Exons are boxed. (C) Table of transmission of mutated alleles for the F0 founders 3, 4 and 10. The numbers of genotyped adult F1 fish, the number of times each different type of mutation (M1-M6) occurred and the counts for WT F1 fish are indicated. (D) Nucleotide sequence of the junction between exon and intron 14 and alignment of the mutations M1 to M6 targeted by the TALEN. Each deleted nucleotides is represented by a dash. The number of deleted bases is reported on the right-hand side of the alignment. M1 was an over-represented mutation (ORM) but was absent out of 5 males and 5 females wild type TU zebrafish (see S3 Fig). The *col6a1* ^*ama605003*^ line was generated from the M3 mutation (star). (E) Results of RT-PCRs on fin clips of heterozygous F1 fish containing one of the corresponding mutations (as indicated). The 298 bp band corresponds to the wild-type allele of the mRNA, the 244 bp band to the mutated allele. RT-PCR from a wild type (WT) fish is shown on the right hand side.

PCRs encompassing the targeted region of *col6a1* were performed using the GoTaq Flexi DNA polymerase (Promega) in standard 50 μl reactions with 1 μl lysate per reaction as template. The primers used were as follows: forward primer TGCCACCATGAAGAAGAGTG and reverse primer TCAGATGTGAGTTGCTCAGAC (gp1, gp2, Fig 3A). The size of the amplified sequence was 246 bp (see Fig 3). Specificity and concentration of the PCR product was verified on a 2.5% agarose gel in 0.5 x TBE containing ethidium bromide at a final concentration of 0.5 μg/mL.

### T7 endonuclease I assay

For the T7 endonuclease I assay, 10 μl of non-purified PCR product (ca. 150 ng) were denatured and re-annealed in a thermocycler by incubation for 10 min at 95°C, 95°C–85°C at a rate of -2°C/s, 85°C–25°C at a rate of -0.3°C/s and then held at 4°C. The re-annealed DNAs were then treated with 5 U (0.5 μl) of T7 endonuclease I (New England Biolabs) upon addition of 1.5 μl buffer 2 (New England Biolabs) and 3.5 μl H_2_O to a final volume of 15 μl. Reaction mixes were then incubated for 30 minutes at 37°C and resolved on agarose gels analogously to untreated PCR products. Mismatches at the targeted region of *col6a1* were expected to result in cleavage of heteroduplexes thus generating two additional fragments of ca. 136 bp and 110 bp.

### Confirmation of mutations by sequencing

To confirm mutations in the *col6a1* gene in mosaic F0 embryos or adult fish positive by T7 endonuclease I assay, PCRs on lysates encompassing the targeted site were cloned in the TA-pGEM-T Easy Vector (Promega) according to the manufacturer’s protocol and transformed in One Shot TOP10 Chemically Competent bacteria (Life Technologies). Upon overnight blue-white antibiotic selection, PCRs were performed on white colonies using the GoTaq Flexi DNA polymerase (Promega) in standard 10 μl reactions. PCR products were purified using a NucleoFast 96 PCR Plate and sent for sequencing by the reverse primer. Sequences were aligned to the reference sequence using the CLC Main Workbench program (Qiagen). Only insertions/deletions ≥ 2 bp were considered as mutations due to TALEN activity in order to eliminate possible errors generated in the amplification process.

For non-mosaic embryos/adult fish of generation F1 or higher, PCRs on lysates were directly purified and sequenced. If needed, TA cloning was performed as described above for sequences containing mixed traces, and sequences of individual alleles obtained.

### RT-PCR and detection of exon 14 skipping of *col6a1*

Total RNA extractions from embryos and fin clips were performed using the TRIzol reagent (Life Technologies) according to the manufacturer’s protocol. Reverse transcription was performed on total RNA using MMLV transcriptase (Promega) and an oligo (dT) 15 primer (Promega) for 1 h at 42°C followed by inactivation of the enzyme for 5 min at 94°C. The cDNA was used as template in standard PCR reactions with GoTaq Flexi DNA polymerase (Promega). The primers used were: forward primer GGTCCAGTCGGTTACCAAGG (in exon 10, sp1, Fig 3B) and reverse primer CAGATGGTCCGTAGTTTCCAGG (in exon 16, sp2, Fig 3B) and the expected size of the PCR product was 298 bp for a WT allele and 244 bp for an allele with exon skipping (see Fig 3). PCR products were resolved on 2.5% agarose gels in 0.5x TBE containing ethidium bromide at a final concentration of 0.5 μg/mL and skipping of exon 14 was confirmed by sequencing.

### Histology and transmission electron microscopy

For hematoxylin-eosin-safran and Masson’s trichrome (Novotec) stainings, muscle samples of 5 mpf fish were fixed in Bouin, embedded in paraffin, and 5 μm-thick sections were performed (Novotec, Lyon, France). Deparaffined sections were then stained with either one of the two coloration methods. For semi-thin sections and TEM procedures, fish at 2-day and 3-week post-fertilization were euthanized in lethal concentration of MS222 according to the animal care regulation, fixed in F1 (2% paraformaldehyde, 2.5% glutaraldehyde), embedded in agar 3%, vibratome-sectioned at 200 μm. Sections were post-fixed in 1% osmium plus 1% potassium ferricyanide, stained with uranyl-acetate en-bloc, dehydrated and embedded in epon (Embed-812, EMS). Three fish of each WT, HT and HM group of 3 mpf were immersed into lethal concentration of MS222 then perfused through the heart with F1 fixative. Two mm surgery blade transversal hand-sections of the whole body, posterior to the intestinal cavity, were embedded in agar 3% and processed as described above. Epon blocs were sectioned with an ultramicrotome (Leica EM UC6) equipped with a Jumbo-histo diamond knife (Diatome). Semi-thin sections (1 μm) were stained with Richardson’s stain for histopathological analysis and imaged with a Leica DMR microscope equipped with a Color View Soft Imaging camera. Ultrathin sections (80 nm) were prepared with the same diamond knife and collected on slim-bar (200 mesh) grid and 1 mm x 2 mm slot grid with pioloform membrane (Agar Scientific) without further staining. Observations were performed using a transmission electron microscope (JEOL 1400). Images were acquired using a post-column high-resolution (11 megapixels) high-speed camera (SC1000 Orius; Gatan) and processed with Digital Micrograph (Gatan). In each fish, at least 3 myomeres were analyzed.

### TUNEL assay for detection of apoptosis

Fish at 3 week post fertilization (wpf) or 3 month post fertilization (mpf) were euthanized in lethal concentration of tricaine, fixed in 4% PFA and sagittal cryosections performed. TUNEL assay was performed with the DeadEnd Fluorometric TUNEL kit (Promega).

### Locomotion analysis

For the locomotion analysis, we used 3 mpf (n = 12, 16 and 11 for WT, HT and HM respectively) and 9 mpf fish (n = 15, 19 and 7 for WT, HT and HM respectively). Fish were individualized in a dedicated behavior room in tanks (8.8 x 11.8 x 4.6 cm for 3 mpf or 18 x 24 x 9.5 cm for 9 mpf). Tanks were placed on an infrared light box (Viewpoint, Lyon, France) and covered with a Plexiglas lid. Fish were placed, one per tank, in 200 mL (3 mpf) or 1 L (9 mpf) of fish facility water at 26°C and left to recover from stress overnight in the experimental setup. No physical, chemical or visual contact was possible between single fish. The light cycle in the behavior room was identical to the one of the fish facility (light switched on between 9 a.m. and 11 p.m.). On the next day, the spontaneous swimming activity of fish was recorded for one hour at noon with a DragonFly2 infrared digital camera (Point Grey Research, Richmond Canada) at 25 frames per second. Fish were tracked by the VirtualDub software (http://www.virtualdub.org) using a tracking plug-in developed by Yves Lhuillier. The total distance swum and the geographic coordinates of each fish in each frame were automatically determined. The distance swum between two consecutive frames was calculated from the geographical coordinates by triangulation. Instantaneous velocities were calculated for each second as the sum of the distances obtained every 25 frames and the maximum value of instantaneous speed was determined for each fish.

### Analysis of speed distribution

The total cumulated time (s) each fish swam at a given speed was calculated for each 1 cm/s increment from the geographical coordinates determined from the video analysis (see above and S7 Fig). The three speed activity profiles (SAP) were defined according to fish body length (bl.), with 3 mpf (about 2 cm) being half the length of 9 mpf fish (about 4 cm), as follows: rest SAP as the cumulated time the fish swam at a speed inferior to 1 cm/s for 3 mpf or 2 cm/s for 9 mpf, (speed < 0.5 bl./s); the moderate SAP as the cumulated time the fish swam at a speed comprise between 1 cm/s to 6 cm/s for 3 mpf or 2 to 12 cm/s for 9 mpf fish (0.5 bl./s < speed ≤ 3 bl./s); the fast SAP as the cumulated time the fish swam at a speed superior to 6 cm/s for 3 mpf or 12 cm/s for 9 mpf (speed ≥ 3 bl./s). The relative proportions of each of the 3 SAP for each genotype were calculated as a percentage for 3 mpf and 9 mpf fish.

### Occupancy of fish in the water column

Fish were conditioned one per tank overnight in tanks of 11.5 x 22.5 x 12.5 cm with 2 L of facility water and then video-recorded at 25 frames per second, in the vertical plane for 30 min. The cumulated time each fish swam in the upper quarter of the tank was calculated from the videos (watched in accelerated mode at speed 4x). Vertical swimming trajectories were calculated from the geographical coordinates of fish tracked analogously to the locomotion analysis. For the diagrams, the position of the fish every 6 sec (1 frame every 150 frames) was plotted through the entire length of the films (30 min).

### Centrophobia analysis

To determine if fish at 9 mpf presented signs of centrophobia, we calculated the time swum by fish in the centre of the tank. For this, the centre was defined as the central horizontal zone, homothetic to the aquarium and covering 50% of the total surface of the tank. To realize this test, the films from the locomotion analysis (see above) were used.

### Respiration rate

Fish were conditioned individually overnight in tanks of 11.5 x 16.5 x 9 cm with 1.5 L of facility water. Then the respiration rate was determined by manually counting the oral/opercular movements of each fish for one minute in the fish facility to minimize stress conditions.

### Statistical analyses

For the behavioral studies on adult fish, data distributions were not tested to determine normal distribution, therefore they were analyzed through nonparametric tests. Chi-square tests were used to compare speed distributions and were performed with R. Mann-Whitney tests were used for all other pairwise comparisons and were performed with Statview. In the behavior analysis Figs (9 and 10), symbols *, ** and **** correspond to p < 0.05, p < 0.01 and p < 0.0001 respectively.

## Results

### Establishment of a stable zebrafish line using TALEN-mediated targeted mutagenesis of the exon 14 / intron 14 junction in the *col6a1* gene

In patients, skipping of exon 14 of the *col6a1* gene encoding the collagen type VI alpha-1 chain has been demonstrated to lead to the onset of BM, although a case of mild UCMD caused by this mutation has also been reported [42]. In zebrafish, exon 14 of the *col6a1* gene (genome assembly Zv9, NCBI *Danio rerio* annotation release 103), is homologous to exon 14 in humans. In zebrafish embryos, injection of morpholinos blocking the splice donor consensus sequence of intron 14 resulted in an in-frame skipping of exon 14 [36]. In the study of Telfer et al. [36], exon 14 is referred to as exon 13 since their report was based on *Danio rerio* genome assembly Zv8. In order to reproduce the skipping of exon 14 in a stable zebrafish line, we targeted the splice donor site of intron 14 with a TALEN directed against the exon/intron junction (Figs 1 and 2A). As natural polymorphism is a hindrance when trying to isolate NHEJ-induced mutations, we checked the targeted locus for polymorphism. The surrounding of the locus was found to be homozygous and to be of the same sequence as reported in the Zv9 genome in all fish tested (fin clip, PCR of 417 bp followed by direct sequencing of 5 males and 5 females non-injected TU, (data not shown). We injected three different doses of the TALEN mRNA pair (200, 400 and 600 pg/pair) at the zygote stage and counted normal, malformed and dead embryos (Fig 2B). The mutation rate as a function of the dose the TALEN mRNA was assessed by T7 endonuclease I (T7 EI) assay using the primers gp1 and gp2 (Figs 2C and 3A). The mutation rate of the three doses was not significantly different (exact two-tailed Fisher’s test) ranging from 5% for the two lower doses to 25% for the highest dose. F0 fish injected with the highest dose (600 pg) were raised to sexual maturity. Only normal embryos were retained for further studies and husbandry. We obtained 10 F0 fertile adult fish and analyzed fin biopsies of each of them for the presence of somatic mutations. The analysis was performed by sequencing of PCR products flanking the target site of the TALEN i.e. amplicons were individualized by TA cloning and 16 to 20 of them were sequenced (S2 Fig). We found that three fish carried somatic mutations at a rate of 5% or higher (fish # 1, 3 and 8; S2A Fig). Such high mosaicism reflects the cumulative effect of the fast development of zebrafish and the relatively high stability of the TALEN mRNA [43]. In these conditions, the TALEN induces stochastically new mutations in different cells at different times (S1 Fig). Indeed, mosaicism is not limited to somatic cells but is found also in the germline.

In the next step, we tested the progeny of each F0 fish for the presence of mutations. Briefly, each injected F0 fish was crossed with a wild type (WT) zebrafish. Of the resulting F1 eggs, 24 were individually lysed and served to amplify a DNA fragment encompassing the TALEN target site. The PCR fragments were then submitted to T7 EI assay. Positive samples were further analyzed by direct sequencing. The analysis of the F1 eggs showed that 7 F0 fish transmitted at least one mutation to their progeny (S2A Fig). One of the fish positive for the fin clip test failed to transmit any mutation. The number of positive embryos per clutch ranged from 1 to 11 out of 24. One over-represented mutation was present in the progeny of all 7 fish but absent from non-injected animals (S2 Fig). Three F0 fish transmitted more than one mutation to their progeny (fish 3, 4 and 10; S2 Fig). We chose to raise to adulthood only the progeny of those three fish.

In a second set of experiments, we obtained 280 F1 adult fish and analyzed a fin clip of each of them by direct sequencing of PCRs flanking the target site (gp1, gp2, Fig 3A) to identify heterozygous mutants (HT). Positive samples were thereafter TA-cloned and sequenced to determine the precise sequence of the mutation. Out of the 280 F1 fish, we isolated 36 adult heterozygous fish carrying one amongst six different mutations (small deletions M1-M6) at the targeted locus (Fig 3B, 3C and 3D). Five of the isolated mutations affected the splice donor consensus sequence at the beginning of intron 14: M1, M2, M3, M5, and M6. Two of those mutations also affected the end of exon 14: M2 and M6. Finally, one mutation, M4, did not affect the splice donor and consisted in a deletion of one nucleotide at the end of exon 14 introducing a frame-shift (Fig 3D).

### Correlation between isolated mutations in the *col6a1* gene and in-frame skipping of exon 14 in the spliced transcript

In order to confirm that the isolated mutations lead to exon skipping, we performed RT-PCR on total RNA from fin clip of F1 fish (Fig 3). We used primers sp1 and sp2 located within exon 10 and 16 of the *col6a1* gene (see Materials and methods and Fig 3B). The expected PCR product size was 298 bp for a wild type allele and 244 bp if exon 14 (54 bp long) was skipped (Fig 3B). In-frame exon skipping was confirmed for four mutations (M2, M3, M5 and M6) by gel electrophoresis of RT-PCR products (Fig 3E) and cDNA sequencing (data not shown). As expected, no exon skipping was observed for mutation M4 (see Fig 3E), however, cDNA sequencing confirmed that mutation M4 does indeed induce a frame-shift (data not shown). More surprisingly, we could not detect any exon skipping allele corresponding to mutation M1 (Fig 3E); similarly, sequencing did reveal no frame-shift either (data not shown). The mutation M1 was absent in the sequence of 10 wild type adults, 5 males and 5 females (S3 Fig). The most probable explanation for this result is nonsense-mediated mRNA decay [44] which is a well-established outcome of some splice site mutations and results in the selective degradation of the mutated mRNA.

As we were in search for an exon-skipping mutation, we decided to establish the M3 mutation as our working fish line (*col6a1* ^*ama605003*^) as we had both male and female F1 heterozygous fish available. This enabled us to generate directly F2 homozygous mutant (HM) fish. All remaining experiments described in the paper were carried out with the *col6a1* ^*ama605003*^ fish line.

### *col6a1* is expressed from embryo to adult in WT and zebrafish mutants

**Fig 4:**
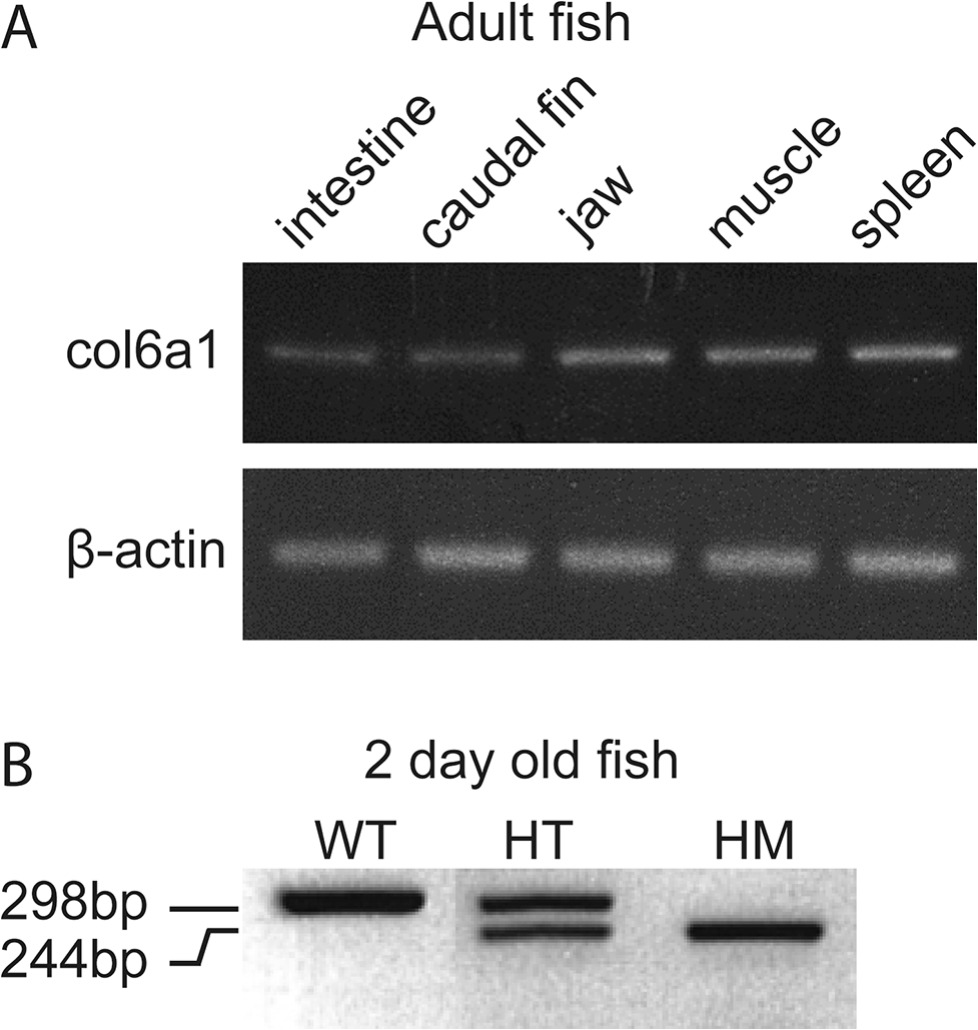
Validation of the presence of *col6a1* mRNA by RT-PCR at different developmental stages in different organs of zebrafish. (A) Confirmation of the presence of *col6a1* mRNA in different organs of adult WT zebrafish: c*ol6a1* mRNA is detected in the electrophoregram of organs from adult fish as indicated at the top of the panel. β-actin is used as internal control. (B) Confirmation of the presence of two forms of *col6a1* mRNA in wild-type (WT) and mutant (presenting exon 14 skipping) zebrafish embryos at 2 dpf from *col6a1* ^*ama605003*^ line. WT fish express only the wild type allele (298 bp); heterozyogous mutant (HT) carry both wild type and mutated allele (244 bp) and homozygous fish (HM) express only the exon-skipped form.

We performed RT-PCR on samples of different organs of adult WT fish that confirmed the ubiquitous *col6a1* expression in intestine, caudal fin, jaw, spleen and in skeletal muscle (Fig 4A). The presence of the *col6a1* transcript in WT as well as mutant fish was confirmed by RT-PCR analysis on whole zebrafish embryos at 2 dpf (Fig 4B).

### *col6a1* ^*ama605003*^ embryos and fry were not morphologically different from WT and fry displayed similar touch-evoked response

Mutant *col6a1* ^*ama605003*^ and WT sibling embryos and fry displayed neither gross phenotype differences nor obvious behavioral changes (data not shown). Birefringence muscle imagery, which is commonly used to identify dystrophic mutants [45,46], was not different in 3 and 4 dpf zebrafish mutants when compared to WT siblings (S4 Fig). Finally, actin staining with fluorescent-conjugated phalloidin did not reveal significant structural alterations of muscle cells in the trunk of 3 dpf mutants and WT siblings (S4 Fig). In order to assess locomotor activity of the fry, the touch-evoked response was measured in 2 dpf mutant fry as previously described [46,47] and did not reveal significant differences between mutants and WT siblings (S5 Fig).

### Age-dependent progressive disorganization of myofibers of *col6a1* ^*ama605003*^ mutant fish without evidence of apoptosis

**Fig 5:**
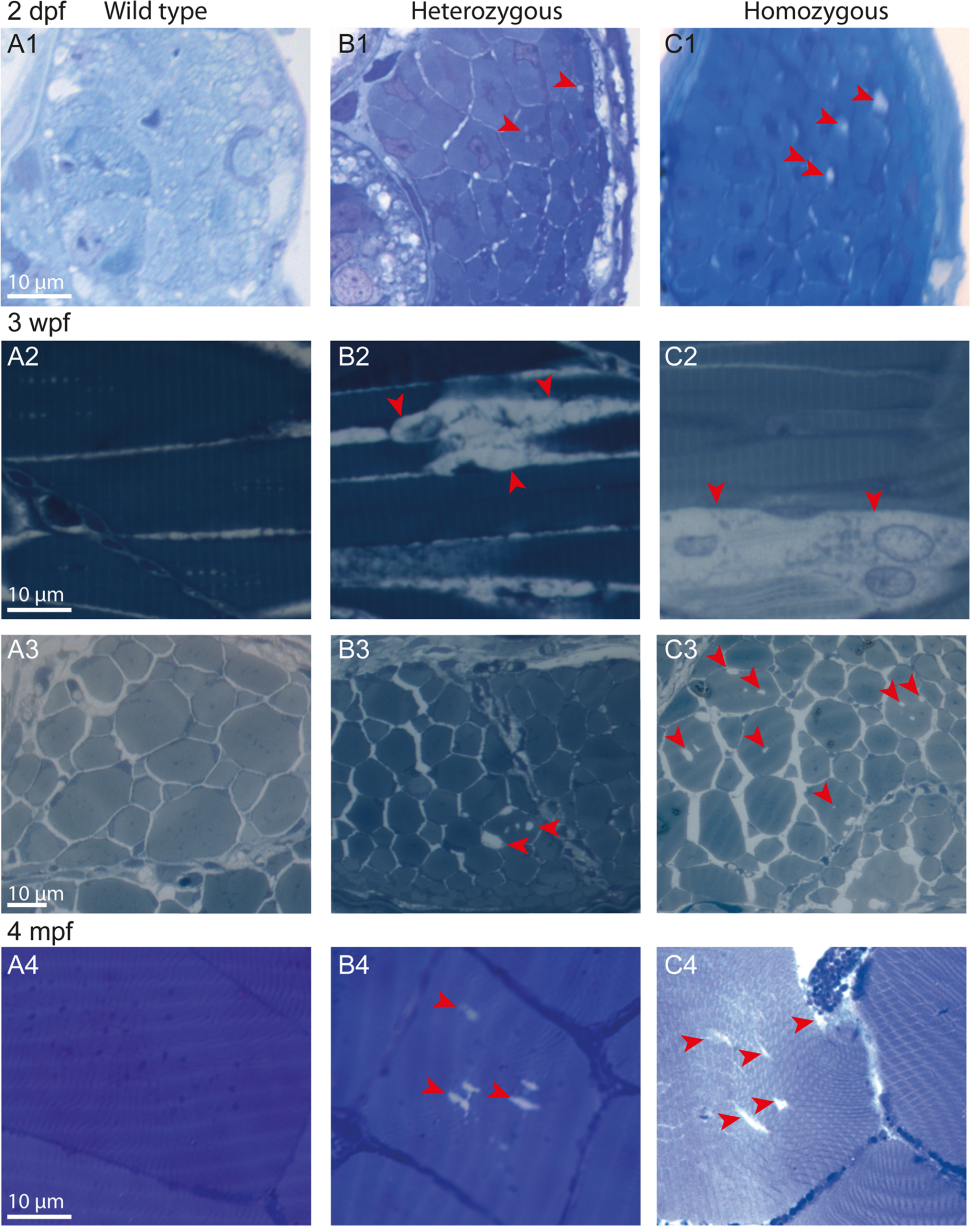
Age-dependent progressive disorganization of muscle fibers of *col6a1* ^*ama605003*^ mutants. Light photomicrographs of Richardson’s stained semi-thin 1-μm sections from wild type (WT, A1-4), heterozygous (HT, B1-4) and homozygous (HM, C1-4) *col6a1* ^*ama605003*^ mutants at 2 days, 3 weeks and 4 months post-fertilization (2 dpf, 3wpf, 4 mpf, respectively). Right from 2 dpf in HT (B1) as in HM (C1) mutants, we observed abnormal vacuoles (red arrowheads) in the centre of some of the muscle fibers. Muscle fibers adjacent to abnormal ones remained similar to the ones WT (A1). At 3 wpf and at 4 mpf, in HT (B2-4) as in HM (C2-4), the number and the size of abnormal vacuoles in myofibers varied from one area to another. Abnormal myofibers with vacuoles were scattered among unaffected myofibers identical to those observed in WT (A2-4). In sagittal sections of muscle from 3 wpf HT we observed breaks in fiber tethering (B2, red arrowhead) or cell in advanced degradation (C2, red arrowheads). Sections were cut according to transversal plane, except A2, B2, and C2 which were sagittal sections. Scale bars, 10 μm.

**Fig 6:**
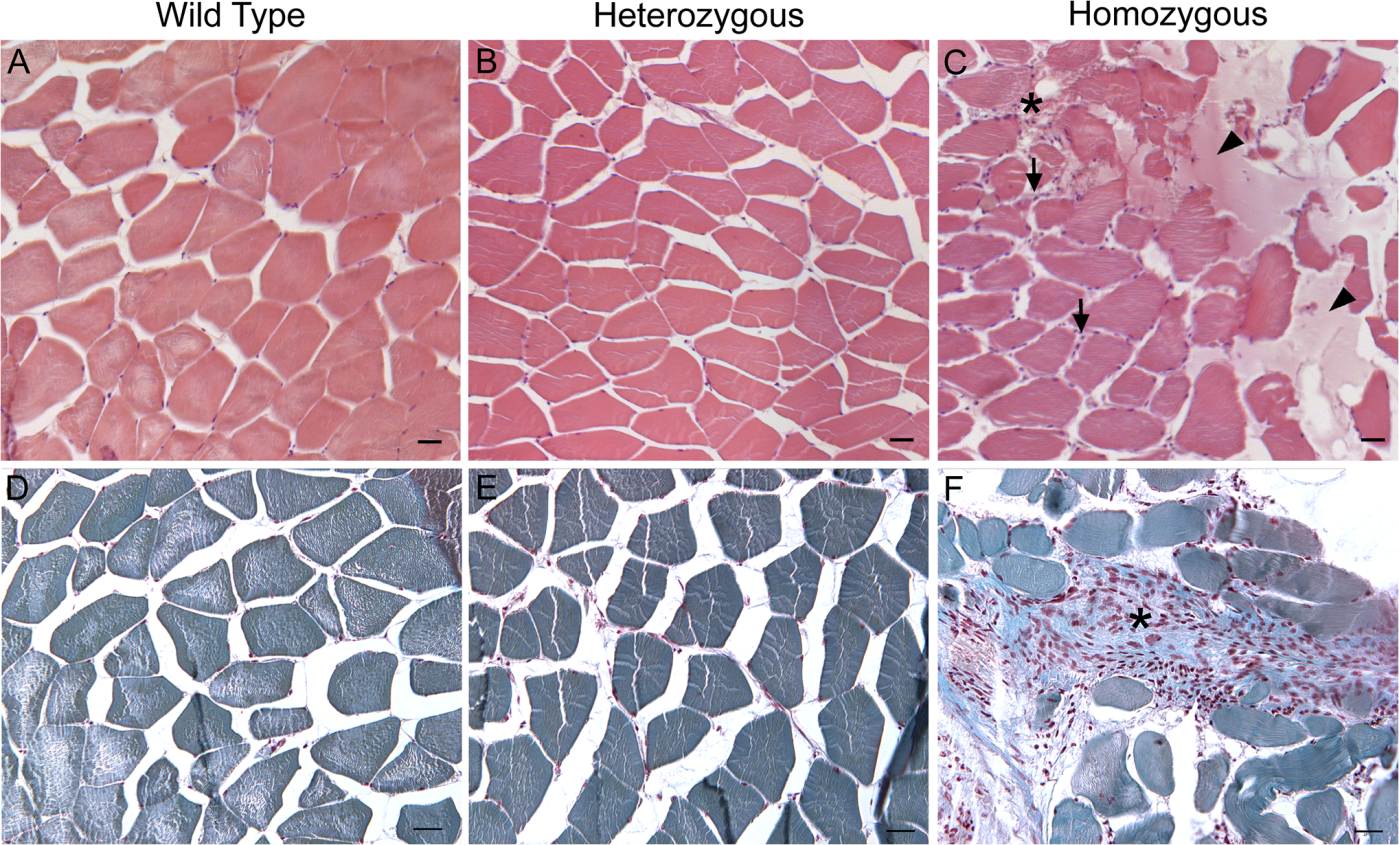
At 5 mpf, in *col6a1* ^*ama605003*^ mutants muscles, the number of nuclei was increased additionally HM muscle showed fibrosis. Light photomicrograph of 5 μm-thick paraffin section of trunk muscle of 5 mpf wild type (WT, A, D), heterozygous (HT, B, E) and homozygous (HM, C, F) *col6a1* ^*ama605003*^ mutants. The section were cut according to transversal plane and stained with hematoxylin-eosin-safran (A-C) or with Masson’s trichrome (D-F) colorations. We observed a slight increase in nuclei in the fibrous septa of *col6a1* ^*ama605003*^ HT (B) and numerous nuclei in HM (C, arrows). Additionally, we observed unidentified amorphous material only in HM (C, arrowheads), and a figure of putative fibrosis (C, star). The Masson’s trichrome confirmed the increasing number of nuclei (violet) from HT (E) to HM (F) as compared to WT (D). This staining also confirmed the presence of large collagen-rich areas most probably marking fibrosis (star, F) that was absent in WT (D) and HT (E). Scale bars 25μm.

Histology of the trunk skeletal white muscle was evaluated by Richardson’s staining from transversal and sagittal semi-thin sections. We studied HT and HM *col6a1* ^*ama605003*^ mutants and WT siblings at 2 days, 3 weeks and 4 months post-fertilization (2 dpf, 3 wpf, 4 mpf). At 2 dpf, we observed a mild disorganization of the skeletal muscle tissue in transverse sections of HT (Fig 5B1) and HM (Fig 5C1) *col6a1* ^*ama605003*^ fry as compared to WT (Fig 5A1): the vast majority of the myofibers were normal but a few of them already presented abnormal intracellular vacuoles. At 3 wpf, the phenotype worsened with noticeable abnormal intracellular vacuoles in the myofibers of mutants (red arrowheads, Fig 5B2-3 and 5C2-3). While the vacuoles were indeed present in myofibers of HT fish (Fig 5B2-3), they were clearly more numerous in the myofibers of HM fish (red arrowheads, Fig 5C2–5C3). At 4 mpf, myofibers with abnormal vacuoles persisted (Fig 5B4 and 5C4). We observed a relatively low number of altered myofibers in the representative sections we analyzed, which reflects the patchy distribution of abnormal myofibers amongst normal ones in mutant fish. In contrast, we were unable to find any abnormal vacuoles in myofibers in any muscle section from the WT fish we analyzed (Fig 5A1-4). Additionally, in *col6a1* ^*ama605003*^ mutants, cell-to-cell contact appeared to be weaker: we observed large gaps between pathological fibers and their neighbours in sagittal section (red arrowheads; Fig 5B2–5C2), although healthy myofibers were in close contact. This was probably due to a greater fragility of mutant myofibers towards the contraction-induced fixation artifact [48]. Generally, the muscular tissue of *col6a1* ^*ama605003*^ mutants appeared more fragile and the myofibers were not tethered to each other as well as in WT. The Richardson’s staining appeared also weaker in the altered cells than in the healthy ones (Fig 5B and 5C). The presence of apoptosis was assessed by TUNEL on cryostat sections of trunk skeletal muscles at 3 wpf and 4 mpf on WT as well as on HT and HM *col6a1* ^*ama605003*^. No obvious difference in TUNEL was found between WT and either *col6a1* ^*ama605003*^ mutant (data not shown). Finally, at 5 mpf, hematoxylin-eosin-safran or Masson’s trichrome stainings of transversal paraffin sections of white muscles were performed to visualize cell nuclei and collagen content respectively. Indeed, *col6a1* ^*ama605003*^ mutant muscles showed a increase in the number of nuclei, most probably due to the presence of numerous fibroblasts, and associated abundant extracellular matrix indicating the development of fibrosis in damaged muscles (violet; Fig 6B, 6C, 6E and 6F). In HM, the increase in number of nuclei was amplified (arrows, Fig 6C) and was associated with the development of areas with accumulation of extracellular material (arrowheads, Fig 6C). Finally, we also observed in HM collagen-rich areas with numerous nuclei (star, Fig 6F) most probably due to fibrosis that was unseen in WT or in HT (Fig 6).

### Ultrastructural defects of muscle fibers in *col6a1* ^*ama605003*^ mutants were already present in embryos and worsened with age

**Fig 7:**
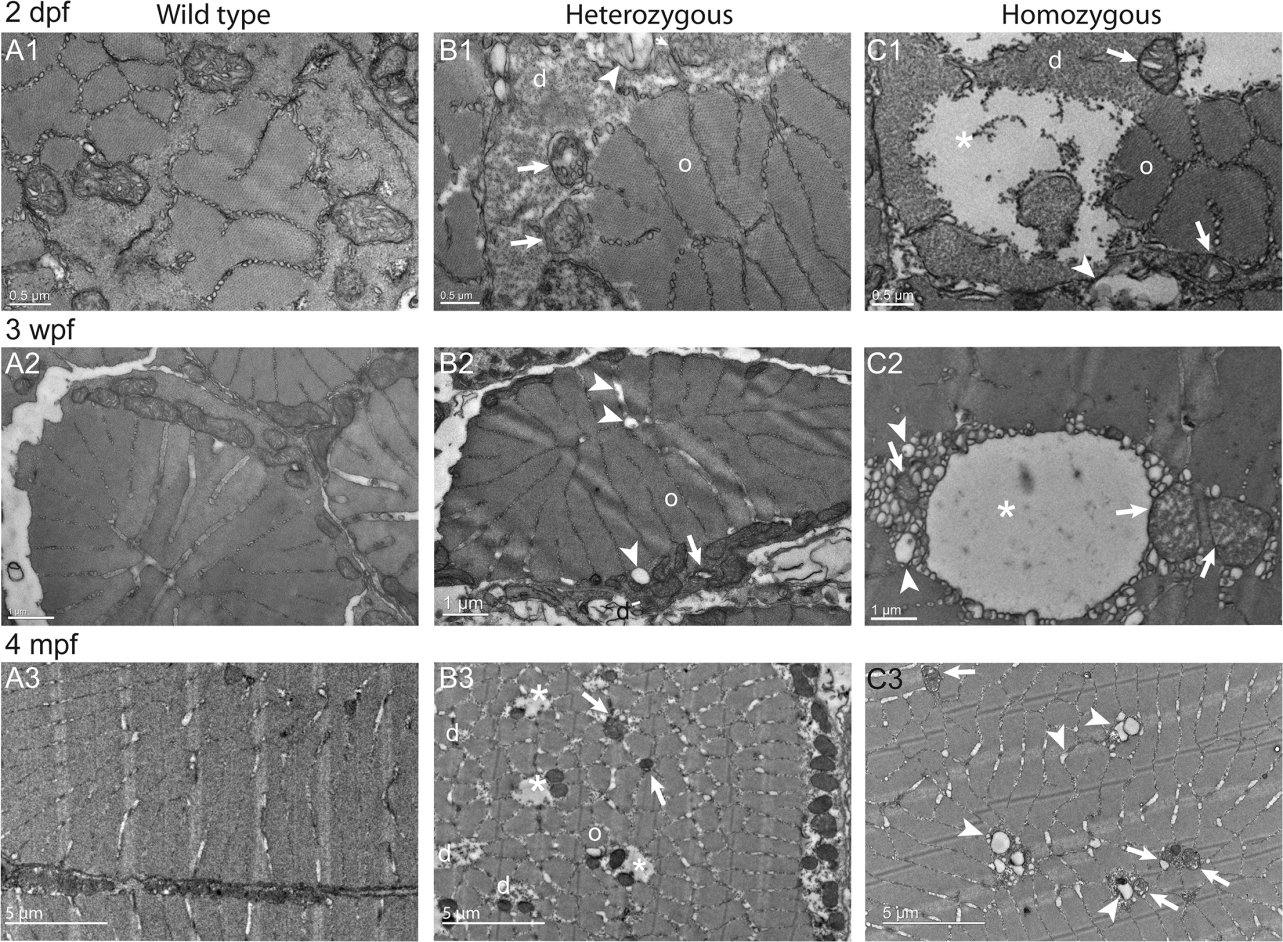
Disorganized Cohnheim’s fields, swollen reticulum and altered mitochondria in *col6a1* ^*ama605003*^ mutant fish muscle. TEM pictures of transversal sections of muscle from wild type (WT, A1-3) and *col6a1* ^*ama605003*^ heterozygous (HT, B1-3) and homozygous (HM, C1-3) mutants at 2 dpf, 3 wpf and 4 mpf. At 2dpf (A1, B1, C1), 3 wpf (A2, B2, C2) and 4 mpf (A3, B3, C3) in HT and HM mutants, we observed in a few muscle fibers, abnormal mitochondria with areas of the matrix devoid of electron dense material (B1-3, C1-3, arrows). In these myofibers, the abnormal mitochondria were often located in close contact with enlarged sarcoplasmic reticulum (B1-2, C1-3, arrowheads). The altered mitochondria often marked the limit between crystal-like organized (o) Cohnheim’s field and pathologic disorganized (d) myofibrils (B1-3, C1-2). In HM (C1), some fibers appeared more affected than in HT, with drastically disorganized (d) areas separated from organized myofibrils (o) with mitochondria having swollen cristae (C1-3, arrows). The sarcoplasm area of the more affected fibers appeared devoid of material (B3, C1-2, asterisks). (B3) In a few muscle fibers of HM, the reticulum appeared drastically dilated (asterisk) with numerous blebs or autophagic vacuoles localized nearby or within myofibrillar disarray foci (C2) that were also in close contact with mitochondria with enlarged cristae (C2-3, arrows). None abnormal mitochondrion, myofibril disorganization or enlarged reticulum was seen in WT (A1-3).

**Fig 8:**
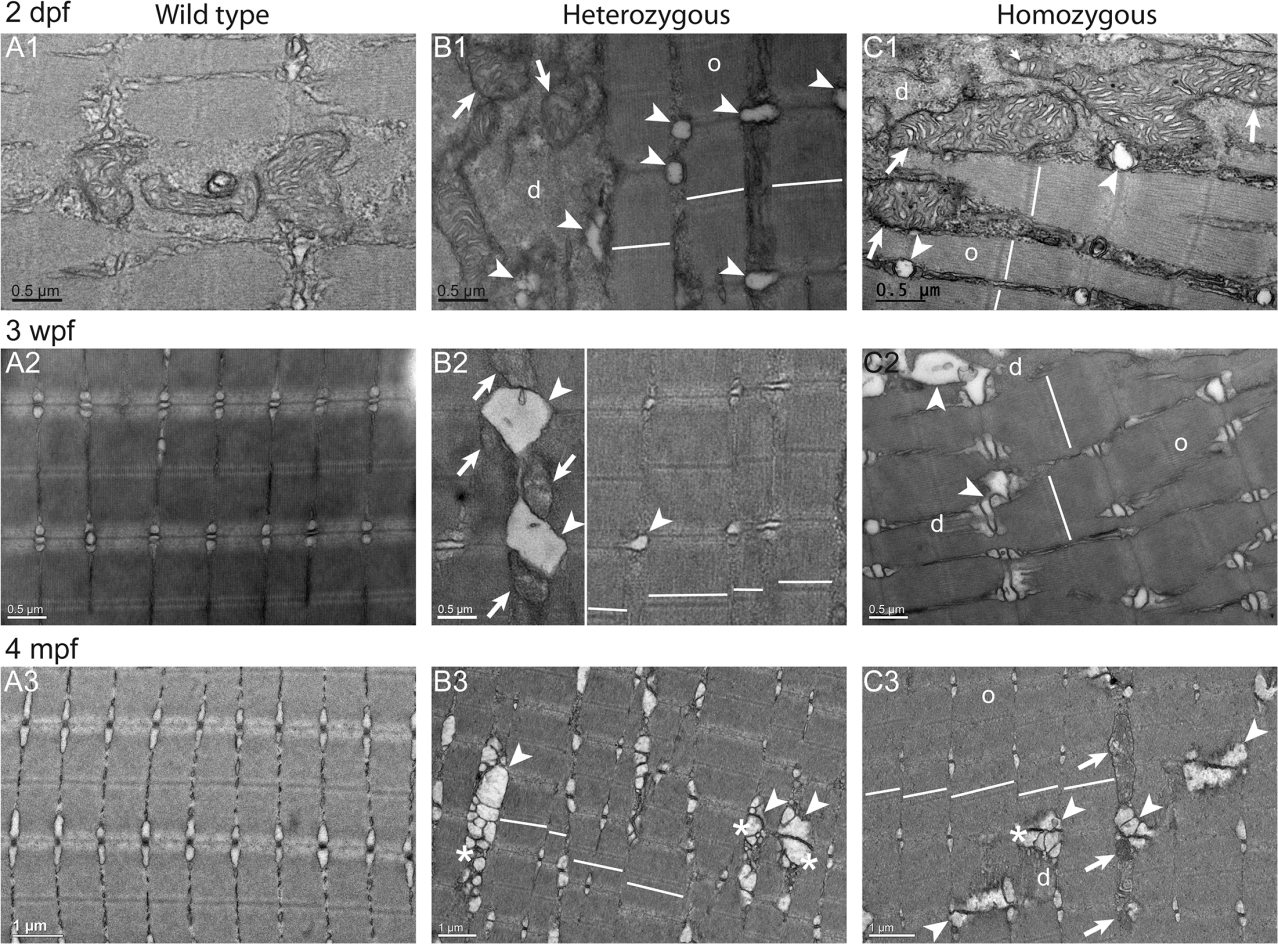
Misaligned sarcomeres in *col6a1* ^*ama605003*^ mutant fish muscle. TEM pictures of sagittal section of muscle from wild type (WT, A1-3) and *col6a1* ^*ama605003*^ heterozygous (HT, B1-3) and homozygous (HM, C1-3) mutants at 2 dpf (A1, B1, C1), 3 wpf (A2, B2, C2) and 4 mpf (A3, B3, C3). As shown in Fig 7 (transversal sections), we observed a swelling of the sarcoplasmic reticulum (B, C, arrowheads), the presence of abnormal mitochondria (B, C, arrows) and vacuoles with membrane blebbing of possibly autophagic nature (B and C, asterisks) in HT and HM mutants at all three ages. In the same myofibre, we also observed disorganized (d) regions amongst still well-organized (o) myofibrils. In sagittal section of HT (B) and HM (C) mutants muscles, the position of the Z-discs and M-bands revealed a conspicuous misalignment of adjacent sarcomeres (white lines). Finally, in HM at 3wpf and 4 mpf (C2-3), the actin filaments in some sarcomeres appeared detached from the Z-line where the vacuole/autophagic vesicles were present (asterisks). No ultrastructural abnormalities as the ones described above were seen in WT (A1-3).

**Fig 9:**
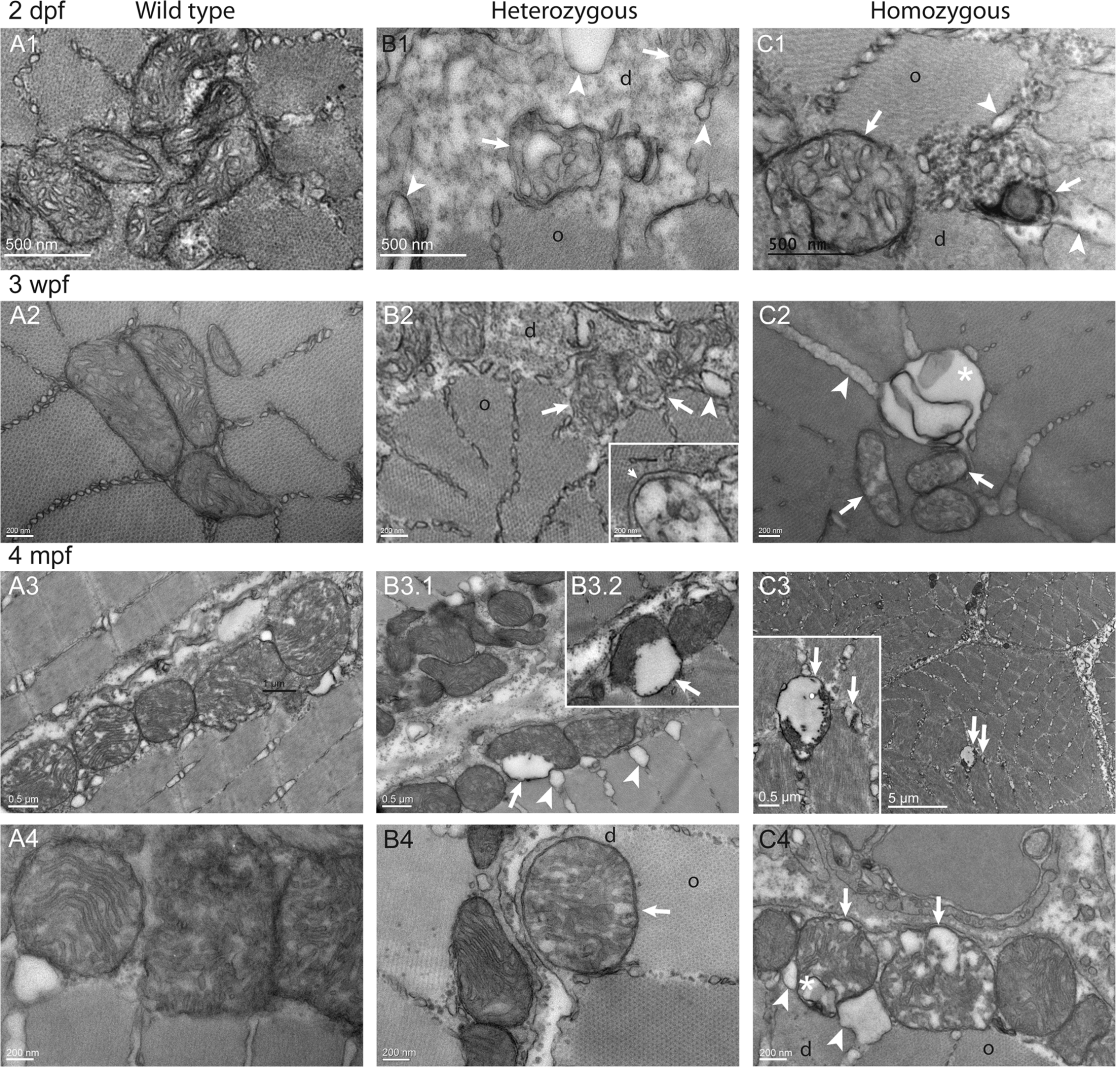
Altered ultrastructure of mitochondria in *col6a1* ^*ama605003*^ mutant fish myofibers. TEM pictures of sagittal sections of muscles from wild type (WT, A1-4), heterozygous (HT, B1-4) and homozygous (HM, C1-4) *col6a1* ^*ama605003*^ mutants, at 2 dpf (A1, B1, C1), 3 wpf (A2, B2, C2), and 4 mpf (A3-4, B3-4, C3-4). In WT, the mitochondria constantly exhibited a dense and well-delineated lamination of the inner membrane and a well-defined outer membrane (A), attesting of the good quality of the fixation-inclusion procedures. In HT and HM muscles, the morphology of mitochondria was diversiform. Similarly to observations in Figs 7 and 8, some mitochondria presented a matrix partly cleared, or even devoid, of electron dense material (B, C arrow) and with severely dilated external membrane detached from the inner condensed membrane (B2-3, C3-4, arrows). These abnormalities were often associated with figures of vacuole/autophagic vesicles (C2, C4, asterisk). In the same area of HT or HM sections, we often observed a normal mitochondrion close to another one exhibiting either swollen cristae (B4, C4, arrows), or even cristae reduced to a few vesicles (B1-2, C2). In a few mitochondria the cristae were even ultra-condensed to a dense core separated from the outer membrane by a vesicle (arrows, B3.1–2, C1). The abnormal mitochondria most often bounded the limits between the crystal-like organized (o) and disorganized (d) myofibrils.

In order to better characterize myofibre defects, we studied transverse and sagittal, ultra-thin sections of muscles from *col6a1* ^*ama605003*^ mutant and WT zebrafish, at 2 dpf, 3 wpf and 4 mpf (Figs 7–9) by transmission electron microscopy (TEM). These results confirm and complete those obtained by light microscopy (Figs 5 and 6) such as the patchy distribution of abnormal myofibers and the presence of abnormal intracellular vacuoles, and finally emphasize structural defects in mitochondria, myofibrils and the sarcoplasmic reticulum (SR).

In transverse ultrathin sections (Fig 7), an obvious disorganization of myofibrils was observed in the outer border of some myofibers, at all ages both in HT and HM *col6a1* ^*ama605003*^ (d, Fig 7B1-3 and 7C1-3 respectively). Large areas of disorganized myofibers were observed (d, Figs 7B and 7C and 8B and 8C). Some myofibers were completely devoid of myofibrils (asterisk, Fig 7C2) even though they were located beside normally organized ones. In these disorganized areas, the mitochondria appeared damaged (see below and arrows Fig 9) and most often bounded the limit between the Cohnheim’s field of well-organized, crystal-like myofibrils and the disorganized ones (Figs 7B and 7C, 8B and 8C). In the same damaged areas, the SR was dilated and disorganized with globular enlargements. Swelling of the SR (arrowheads, Fig 7B1-2 and 7C1-3) was particularly obvious at the level of the terminal cisternae indicative of a possible defect in the excitation-contraction coupling. The section in myofibers of WT sibling presented consistently organized Cohnheim’s field, mitochondria with finely defined cristae and regular SR (Fig 7A1-3).

In sagittal ultrathin sections (Fig 8), the *col6a1* ^*ama605003*^ mutants showed another striking defect. At all ages, the sarcomeres were misaligned both in HT (broken lines, Fig 8B1-3) and HM (broken lines, Fig 8C1-3). Within some myofibers, the Z-discs and M-lines of two adjacent sarcomeres were not aligned in *col6a1* ^*ama605003*^ mutants, contrary to those of WT zebrafish (Fig 8A1-3). Moreover, the cisternal dilations of the SR appeared more conspicuous in the sagittal plane than in the transverse sections. In fact, the whole triads were completely disorganized in mutants with abnormal swelling of SR terminal cisternae and T-tubules (arrowheads, Fig 8B and 8C). Mitochondria with swollen cristae were also found within the myofibrillar disarray foci (arrows, Fig 8B1 and 8C1). All the healthy mitochondria as seen in WT muscle, displayed properly delineated outer and inner membrane with well-disposed and defined cristae (Figs 7A1-2, 8A1 and 9A1-4) indicating well-preserved ultrastructure and excluding fixation artefact. These characteristics of mitochondria in good condition were absent in the myofibrillar disarray foci in HT as in HM *col6a1* ^*ama605003*^ mutants from all ages (arrows, Figs 7–9). Damage onset appeared to begin with a swelling of the mitochondria and the cristae (arrows, Fig 8B1 and 8C1) then to evolve to a complete absence of matrix, with the detachment of the outer mitochondrial membrane from the inner membrane and ending with the hyper-condensation of the internal membrane (arrows, Fig 9B1-2). In what seemed to be an intermediate state, the mitochondria were slightly swollen and the cristae appeared blurred (arrows, Fig 9C1). In these areas, vesicles that resembled autophagic vacuoles or drastically enlarged T-tubules were frequently observed in close contact with altered mitochondria (arrowheads, Figs 7B3 and 7C1-2 and 8B1, 8B3 and 8C2-3). The altered mitochondria were most often located between organized and disorganized myofibrils and, for some of them, in direct contact with enlarged SR (arrows, Figs 7B1, 7B3 and 7C3, 8B2-3 and 8C2-3, 9B1, 9B3.2, 9C3 and 9C4). Finally, at 3 wpf, some myofibers presented sarcoplasmic blebs (S6A Fig) and a few ones showed the typical characteristics of dying cells (S6B Fig).

Altogether, our observations showed that muscle fibers of both HT and HM *col6a1* ^*ama605003*^ displayed disorganized myofibrils, enlarged SR, altered mitochondria morphology and misaligned sarcomeres. The defects started at embryonic stages and worsened as the fish aged. None of these alterations was observed in WT muscles. HM presented a more severe phenotype than HT as expected for a co-dominant mutation. Moreover, we found dying cells only in HM fish and only from 3 wpf onward. Most often, these alterations had a patchy pattern as they were observed in a few myofibers located within undamaged muscle regions. The presence of dying cells only in 3 wpf and 4 mpf HM fish emphasizes the progressive muscle alteration observed in the *col6a1* ^*ama605003*^ homozygous context.

### The *col6a1* ^*ama605003*^ mutants present symptoms of hypoxia at 9 months

Histopathological and TEM imagery showed that muscle tissue, myofibre ultrastructure and mitochondria were clearly altered in *col6a1* ^*ama605003*^ mutants, yet we did not observe any obvious locomotor impairment up to the age 3 mpf. Therefore we studied locomotion in older mutants at 9 mpf. HT, HM and WT siblings were placed individually into tanks and video-recorded in horizontal plane in a free swimming context. Briefly, after transfer into the experimental set-up, the fish were left overnight to recover from stress in a dedicated behavior laboratory (see Materials and methods). On the next day, the fish were video-recorded and the impact of their genotype on swimming behavior was analyzed.

**Fig 10:**
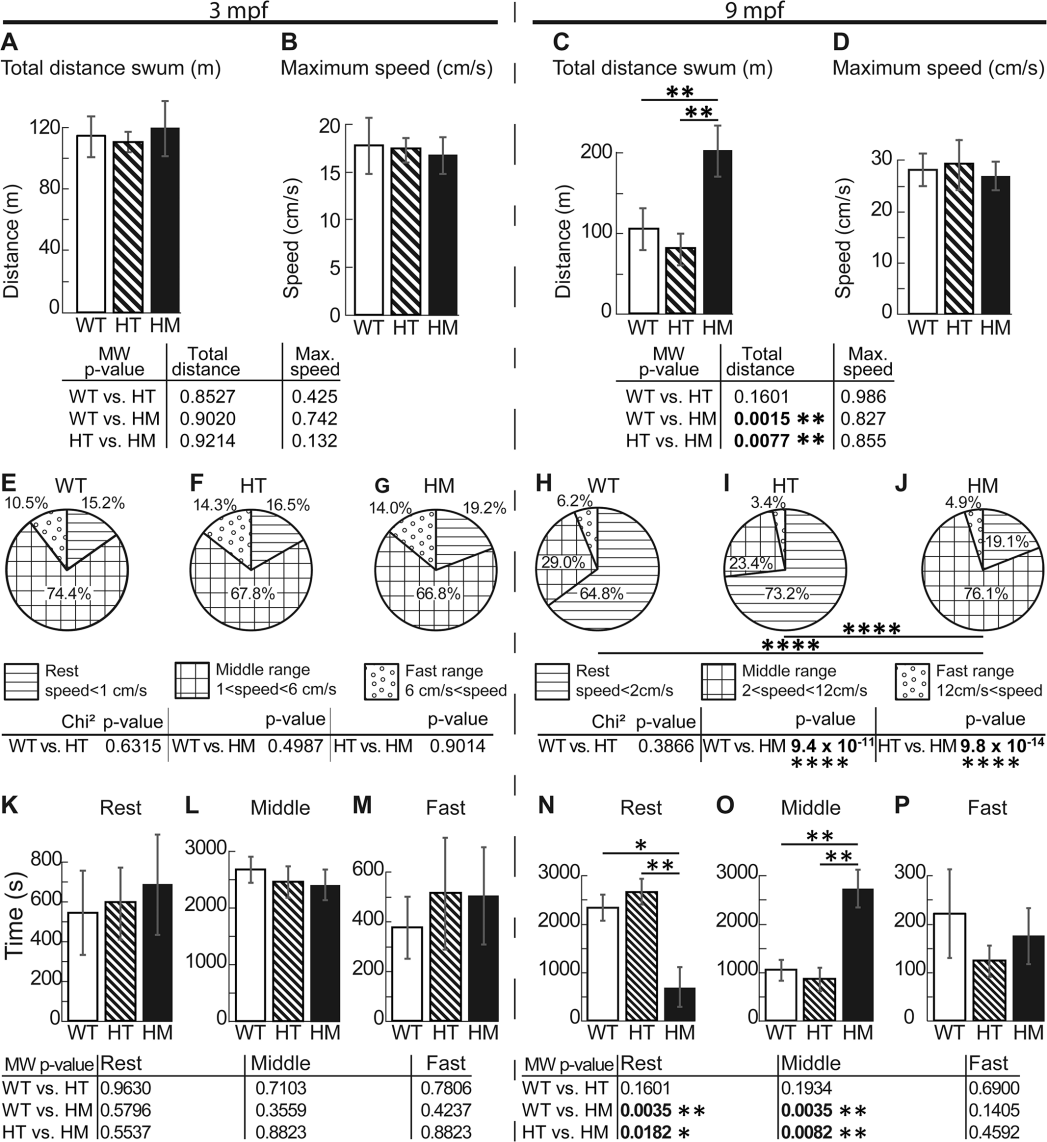
The 9 mpf *col6a1* ^*ama605003*^ HM fish swam around twice the distance of WT and HT fish. We video-recorded free-swimming WT, HT and HM *col6a1* ^*ama605003*^ fish at 3 mpf and 9 mpf for one hour in the horizontal plane, then we calculated by triangulation the total distance swum (A, C) and the maximum instantaneous speed (B, D). (A) At 3 mpf, there was no significant difference in the total distance swam between WT and HT, between WT and HM or between HT and HM. (C) At 9 mpf, there was a significant difference in the total swum distance between WT and HM (C, p-value 0.0015) and between HT and HM (C, p-value 0.0077), but not between WT and HT, indicating the late development of impairment in HM. There was no difference in the maximum speed between genotypes at the two ages studied (B, D). (E-J) We represented in pie-charts the speed activity profile (SAP) distributions in percentage of 3 classes and according to the fish bl. i.e. rest SAP (horizontal lines, speed slower than 1 cm/s for 3 mpf, E, and 2 cm/s for 9 mpf, H), middle range SAP (squares, speed comprise between 1 to 6 cm/s for 3 mpf, F and 2 to 12 cm/s for 9 mpf, I) and fast SAP (open dots, speed superior to 6 cm/s for 3 mpf, G and to 12 cm/s for 9 mpf, J). This representation allowed us to show that at 9 mpf, there is a highly significant difference between WT versus HM (H vs. J, p-value 9.421x10^-11^) and between HT and HM (I vs. J, 9.83x10^-14^). We further analyzed in a pairwise manner the histograms of the time (s) fish swum in the 3 SAP classes (speed distribution) described above for 3 mpf (K, L and M for rest, middle and fast SAP respectively) and 9 mpf (N, O and P for rest, middle and fast SAP respectively). This analysis showed that there was no difference in SAP within any of the 3 mpf groups. But the analysis showed that 9 mpf HM swam significantly more time in middle range SAP at the expense of their resting time i.e. in the rest SAP the difference is significant between WT and HM (N, p-value 0.0035); between HT and HM (N, p-value 0.0182). In the middle range SAP, the difference was significant between WT and HM (O, p-value 0.0035) and between HT and HM (O, p-value 0.0082). For 3 mpf fish, n = 12, 16 and 11 for WT, HT and HM respectively; for 9 mpf fish, n = 15, 19 and 7 for WT, HT and HM respectively. For each histogram and pie-chart, we performed either a Mann-Whitney (MW) or Chi-square test respectively, with *, ** and **** indicating p-values of <0.05, 0.01 and 0.0001 respectively.

**Fig 11:**
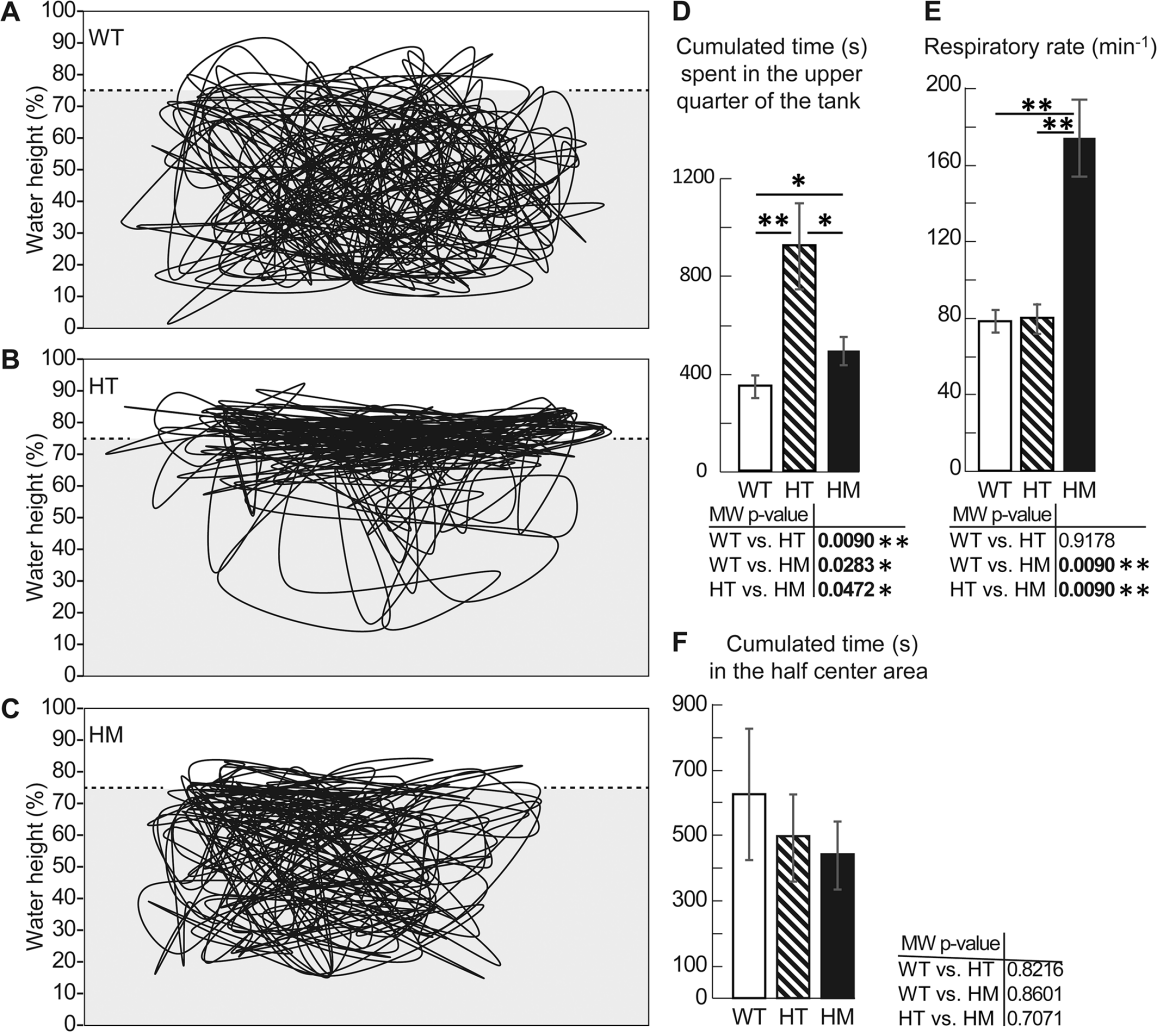
The 9 mpf *col6a1* ^*ama605003*^ mutant fish presented a hypoxia behavior. We video-recorded for 30 min (25 frames/sec) and tracked the swimming trajectories in the vertical plane of WT, HT and HM *col6a1* ^*ama605003*^ fish at 9 mpf in a tank. The position of the fish every 6 sec (1 frame every 150 frames) was plotted. A representative trajectory of each genotype is presented, WT (A), HT (B) and HM (C). From these videos (n = 5 of each genotype) the cumulated time fish swam in the upper quarter of the tank (D) was calculated and was significantly different between WT and HT (p-value 0.009), WT and HM (p-value 0.0283) and HT and HM (p-value 0.0472). HT and HM mutants spent visibly more time in the oxygen-richer part of the tank. The respiratory rate (oral/opercular movements, min^-1^) was determined for mutants and controls, n = 5 (E) and this rate was significantly different between WT and HM (p-value 0.009) and HT and HM (p-value 0.009). As a test for centrophobia, we measured the time fish spent in the centre of the tank in the horizontal plane. The centre corresponds to the half centre area of the tank. There was no difference of occupancy between the three genotypes. n = 15, 19 and 7 for WT, HT and HM respectively. For each histogram, a Mann-Whitney test was performed; *, ** indicate p-values of < 0.05 and < 0.01 respectively.

At 3 mpf, no significant changes were observed between WT and *col6a1* ^*ama605003*^ mutant fish when the total distance swum or the maximum swimming speed (Fig 10A and 10B) was measured. All statistical analyses are indicated in the corresponding tables in Figs 10 and 11. At 9 mpf, maximal instantaneous speed remained similar between genotypes (Fig 10D). Strikingly, however, HM fish swam significantly longer distances, roughly double those swam by WT and HT (Fig 10C; p-value WT vs. HM, 0.0015; HT vs. HM, 0.0077, Mann-Whitney, MW). This result was rather unexpected since HM fish had worse myofibre defects than HT. Thus, we further analyzed the distribution of instantaneous speed from 1 to 21 cm/s of 3 and 9 mpf mutants and WT sibling fish (see S7 Fig). Since the average fish body length (bl.) of 3 mpf (about 2 cm) is the half of the bl. of the fish at 9 mpf (about 4 cm), we defined three classes of speed activity profile (SAP) according to the fish bl.: rest, middle range and fast SAP. Rest SAP was defined as speed below 0.5 bl./s, middle range SAP as speed ranging from 0.5 to 3 bl./s and fast SAP as speed greater than 3 bl./s (see Materials and methods and Fig 10E–10J).

Irrespective of their genotype, all 3 mpf fish groups displayed the same distribution between the three classes of SAP: fish swam roughly 70% of the time at speeds within the middle range and about 15% of the time at speeds within the rest or fast range respectively (Fig 10E–10G). Note that the distribution of instantaneous speed was different between 3 and 9 mpf fish (S7 Fig), thus SAP should only be used to compare free swimming behavior between fish of the same age. At 9 mpf, HT and WT displayed similar SAP: they swam most of the time at speeds within the rest range (~70% of the time), most of the remaining time at speeds within the middle range (~25% of the time), and a little time in fast speed (~5% of the time; Fig 10H and 10I). However, 9 mpf HM fish displayed a strikingly different swimming behavior than their HT and WT siblings (Fig 10J). Thus, HM fish at 9 mpf rested much less (~5% of the time) and spent most of their time swimming at middle range speed (~75% of the time). This difference in SAP was highly significant between WT and HM (p-value 9.421 x 10^−11^, Chi-square) and between HM and HT fish (p-value 9.83 x 10^−14^, Chi-square, Fig 10H–10J). Thus to better analyze this difference in SAP, we further compared in a pairwise manner, the corresponding histograms of swimming speed distributions for the three genotypes in each age group. At 3 mpf, there was no difference between mutant and control fish (Fig 10K–10M). But in the 9 mpf group, HM fish swam significantly more time at middle range activity than WT and HT fish (HM vs. WT p-value 0.082 and HM vs. HT p-value 0.0035, MW, Fig 10O) at the expense of time spent at rest speeds (HM vs. WT p-value 0.0182 and HM vs. HT p-value 0.0035, MW, Fig 10N). Since this type of increased activity or erratic behavior may indicate potential problems in oxygen intake [49] or suffering [50,51], we performed further studies on the respiration behavior of 9 mpf *col6a1* ^*ama605003*^ mutant fish.

To this end, we first tracked the trajectory in the vertical plane of single fish (see Materials and methods). We showed that *col6a1* ^*ama605003*^ mutant fish mostly swam in the upper part of the water column where oxygen is more abundant and defined by Kramer [52] as aquatic surface respiration (ASR; Fig 11A–11C). Hence, HT and HM mutant fish spent a significantly longer time in the 25% upper part of the tank than their WT siblings (HT vs. WT, p-value 0.0009, MW; HM vs. WT, p-value 0.0283, MW; Fig 11D). But unexpectedly, the cumulated times swum in the 25% upper part of the tank for HT fish was significantly longer (p-value 0.0472, MW) than the one of the HM (Fig 11D). Indeed, the main types of behavioral response of fish to decreased oxygen availability in water are activity profile changes, increase in respiration rate and surface respiration, and changes in vertical or horizontal habitat [49,53]. As a matter of fact, 9 mpf *col6a1* ^*ama605003*^ HM displayed a dramatic increase in respiration rate (Fig 11E) that is considered a direct response to hypoxia [53–55] i.e. the oral/opercular movement rate of HM fish was significantly higher than that of HT (p-value 0.0090) and WT (p-value 0.0090). There was no significant difference between the respiratory rate of WT and HT *col6a1* ^*ama605003*^ mutant fish. Since centrophobia is a stress index [56,57], we checked if the difference in respiratory behavior was due to stress by measuring the occupancy of the centre of the tank in the horizontal plane. We showed that there was no significant difference in occupancy of the centre of the tank between WT, HT and HM fish groups (Fig 11F). Therefore, we concluded that increased respiratory rates in HM were most probably a physiological response to hypoxia rather than to an increase in stress or anxiety.

Taken together, our data showed that as early as 2 dpf, *col6a1* ^*ama605003*^ mutant fish present alteration of muscle tissue with ultrastructural alteration of SR and of mitochondria. These histological defects progressed slowly in ageing 3 mpf fish, showing clear signs of muscle fibrosis at 5 mpf without any obvious behavioral changes at this age. Then, only at 9 mpf, *col6a1* ^*ama605003*^ fish displayed swimming behaviors typical of hypoxia. Interestingly, the observation of these simple physiological parameters in the behavior of free moving 9 mpf fish clearly discriminates between WT and *col6a1* ^*ama605003*^ mutants. Finally, the severity of the observed phenotype increased from HT to HM exemplifying the co-dominant character of the *col6a1* ^*ama605003*^ mutation.

## Discussion

### A TALEN designed for an exon skipping disease model

We generated several *col6a1* mutant zebrafish lines using a TALE nuclease. To the best of our knowledge, this is the first example in zebrafish which shows that template-independent NHEJ mediated repair can be used to produce an exon skipping mutation. In the zebrafish egg, the majority of generated double-strand breaks (DSB) are repaired by the error-prone NHEJ pathway rather than by homology directed repair [58]. The use of a template-independent method is thus expected to lead to high efficiency of splice site modification similar to NHEJ mediated gene knock out. Short insertions or deletions generated within essential splice sites, are expected to almost invariably modify the splicing. Indeed, the analysis of the various mutations isolated in this study showed that four mutations out of six were located within the splice donor consensus sequence of intron 14 of the gene *col6a1* and induced exon skipping. However, one mutation situated into the splice consensus sequence did not generate mRNA with skipping of exon 14 (M1). There is no easy explanation why mutation M1 (Fig 3) failed to induce exon skipping while M3 (and M2, M5 and M6 for that matter) did so. M1 is clearly over-represented amongst the isolated mutations. This mutation was absent of 10 non-injected wild type TU fish tested (S3 Fig). Similarly, we failed to detect the mutated allele in fin clip of most of the founders (S2A Fig). Hence, parental polymorphism seems unlikely. Sequencing artefact is unlikely as the fish transmitting the M1 mutation were detected using T7 endonuclease assay prior sequencing. Therefore, it seems the M1 mutation is actively favored in our fish. In fact, the total absence of mutated mRNA in the M1 fish line may provide the best explanation for M1 over-representation. Indeed, the absence of mutated mRNA is consistent with a mechanism of nonsense-mediated decay of the mutated mRNA form [44]. The resulting phenotype, a reduced capability to produce Col6a1 protein, would be somehow milder than the structural defects introduced by the other mutations. Thus, M1 mutation might avoid most of the counter-selection which hinders the deleterious co-dominant mutations. Consequently, its apparent prevalence would increased.

Other outcomes of splice site mutations are possible such as the activation of alternative splice sites upon deletion of the normally used ones. Thus, it is important to check that the isolated mutation indeed induces the expected defect. Conversely, different mutations can produce the same exon skipping as our results showed (see Fig 3D). Indeed, any mutation targeting one of the two first nucleotides of the intron can induce the skipping of the preceding exon. Hence, targeting a splice site is as easy as realizing a knock-out with a TALE/CRISPR-Cas9 nuclease. Since many human genetic diseases are caused by splicing defects, this simple and efficient method will help to generate animal models for these diseases [44,59–61]. Application of this strategy may also be useful to induce skipping of defective exons as therapy for other diseases [62,63].

Genotyping of injected animals and their progeny in order to find a heterozygous mutation carrier may be a long and taxing procedure. Most frequently, only a few F0 and F1 animals are analyzed. Mainly due to the mosaicism in F0 and limited sample size, mutations found in a F0 fish may be different from those found in its progeny (S1 Fig). In our study, we detected somatic mutations in only three fish out of ten and from those three only two transmitted one or several mutations to their progeny. Conversely, five fish which were negative for somatic mutations, transmitted germline mutations to their progeny. In zebrafish, soma and germ-line cells are isolated from each other early in development. Consequently, somatic and germ line nuclease-generated mutations derive most frequently than not from independent events. Hence, finding a mutation in the fin clip of a F0 fish is hardly indicative of the presence of mutations in its germ line. Direct screening of the F1 eggs should be preferred to screen for mutations.

### Sarcoplasmic disarray and mitochondrial damage in *col6a1* ^*ama60500*^ muscles

The *col6a1* ^*ama605003*^ stable line we generated, allowed us to study at the functional and histological level collagen VI-deficient fish up to 9 mpf for the first time. The *col6a1* ^*ama605003*^ zebrafish mutants exhibited histological and ultrastructural muscle alterations identical to the ones observed in muscle cell cultures [64–67] and biopsies [67,68] from patients with collagen VI deficiency, in mice [30–35], as well as in the transient zebrafish model generated by morpholino-based knock-down [36,37].

The accumulation of extracellular matrix we observed in transversal section of white muscles (Fig 6) had the typical figure of fibrosis. The presence of fibrosis, essentially composed of collagen, a characteristic of all muscular dystrophies [69], is present in UCMD patient biopsies [23,67] and was also described in the *Col6a3* deficient mouse model [34]. At the ultrastructure level (Figs 7–9), the observed alterations included disorganized myofibrils, conspicuous abnormal dilation of the SR, terminal cisternae and T-tubules in the triads, abnormal mitochondria and misaligned adjacent sarcomeres. These ultrastructural muscle alterations match the clinical manifestations of BM [67,68,70] or UCMD [67,71] and were also observed in the muscles of the *mdx* mouse model of Duchenne muscular dystrophy [72] as well as in damaged muscle of exhausted athletes [73]. Moreover, the ultrastructural alterations we have observed from fry to young adult fish mutants are characteristic for the early ages of collagen VI-related myopathies in humans [67,68,70]. These findings suggest that our fish line is a good model to study the implication of mitochondria and SR in these diseases, which have not been studied extensively.

In our *col6a1* ^*ama605003*^ mutant line, we did not detect increased levels of apoptosis by TUNEL. Our data are in agreement with findings in other animal models with collagen VI deficiency [34], although in *col6a1* ^-/-^ mice, apoptosis was described as a hallmark of the model [31] and it was also present in the transient zebrafish morphants with exon skipping [36]. Nevertheless, we observed dying myofibers most frequently in mutants at 3 wpf and 4 mpf (S6 Fig). Our observation of the presence of autophagic vesicles associated with the aggregation and ultra-condensation of mitochondria together with the absence of TUNEL-positive cells in mutant zebrafish suggest that *col6a1* ^*ama605003*^ deficient muscle cells do not generally die of apoptosis. However, further studies will be necessary to determine whether autophagy is altered in some way in the *col6a1* ^*ama605003*^ zebrafish mutants and could be stimulated as has been described in *Col6a1* ^-/-^ mouse models [31,38,74], since collagen VI can modulate autophagic signalling pathways [75]. Studies on animal models [36,38,76] and clinical trials on patients [65] strongly suggest that activation of autophagy and inhibition of the PTP of mitochondria should be considered as potential treatments for diseases caused by defects in collagen VI.

Moreover, we observed a general increase of muscle tissue alterations in HM compared to HT e.g. dying cells were observed at 3 dpf and 4 mpf in HM but not in HT and increased respiratory rate was observed only in 9 mpf HM. This allele-dependent gravity of the phenotype is consistent with a co-dominant inheritance and correlates with a protein alteration mechanism. This loss of function originates from perturbed assembly and secretion of collagen VI, which reduces the amount of functional protein in the extracellular matrix [4,33] and is probably responsible for the greater fragility of the muscle tissue to contraction-induced stress. The co-dominant mode of inheritance, together with late locomotor/hypoxia phenotype development is reminiscent of observations in patients with BM [4,17].

We also demonstrated an increase of phenotype severity with ageing at the histological ultrastructural and functional levels. In this regard, the *col6a1* ^*ama605003*^ zebrafish line allows the study of symptom progression and muscle alterations in ageing fish. We should emphasize the significance of a fish model which allows studying alterations in ageing muscle in analogy to health deterioration observed in some ageing patients with collagen VI deficiency [21,28,26,71].

### Hypoxia response behavior in aging *col6a1* ^*ama605003*^ fish, a possible link between collagen deficiency, mitochondria damage and respiration

The functional studies on the *col6a1* ^*ama605003*^ mutant line showed no alterations in normal touch-evoked response in 2 dpf fish fry (S5 Fig) and in swimming behavior of young 3 mpf adults. With age progression, at 9 mpf, we observed three obvious changes in the behaviors of mutants. First, compared to WT and HT, HM fish swam significantly more time in the range of moderate SAP, spending much less time in the rest range (Fig 10 and S7 Fig). Secondly, compared to WT and HT, HM fish had a significant increase in respiratory rate (Fig 11). Finally, 9 mpf HT and to a lesser extent HM fish swam preferentially in the upper part of the tanks where oxygen, diffusing from air, is more concentrated (Fig 11). Moreover, beside their preferred aquatic surface respiration, the behaviors of HT and HM fish were different: the HT dramatically increased erratic swimming and the HM modified their swimming behavior slightly but doubled their respiratory rate. These observations suggested a suffering behavior [50,51] or more specifically hypoxia as they are consistent with reports of increased spontaneous locomotor activity in fish exposed to oxygen deprivation [49,52,53,77]. This conclusion is further supported by the increase in respiratory rate observed in HM as this has been described for fish lacking oxygen [49,55,78,79]. Finally, these behavior changes observed in *col6a1* ^*ama605003*^ mutants were not induced by anxiety (Fig 11) since the mutant fish did not display thigmotaxis, a centrophobic stress behavior [57]. Altogether these elements indicate the presence of a respiratory adaptive response in *col6a1* ^*ama605003*^ mutant fish with a more pronounced phenotype in HM. Indeed, while the change in swimming behavior seems to be sufficient for HT to compensate for hypoxia, the more severely affected HM fish needed additionally to increase their respiration rate. Since collagen VI is expressed in gills [80], the expression of the modified Col6a1 protein might provoke defects in the structure or the function of this organ resulting in an altered intake of oxygen. Alternatively, the reason for mutants to suffer from hypoxia might be attributed to the presence of degenerating mitochondria, which would interfere with the proper cell respiration.

Moreover, interactions between hypoxia and collagen expression have been reported; for review see Salminen et al. [81]. In normal conditions, collagen is stabilized by propyl and lysyl hydroxylation hydrolases (PHD1-3) of the 2-oxoglutarate-dependent dioxygenases (2-OGDO) family, the functions of which are oxygen dependent. PHD1-3-related, non-epigenetic pathways are linked to the hypoxia inducible factors (HIF) which are in turn targeted by PHD1-3. Thus, oxygen controls these pathways through the PHDs that are oxygen sensors [81]. In normal normoxic conditions, PHDs are active and HIF are degraded, whereas in hypoxia PHD are inactive and HIF accumulate inducing the expression of 300 genes involved in the response to oxygen deprivation.

In turn, mitochondria play a key role in many diseases e.g. neurodegeneration, cancer, heart and muscular diseases [82] to cite a few. Here, we showed that mitochondria are probably one of the first organelles altered by the *col6a1* mutation in myofibers. Actually, on one hand the alteration of mitochondria observed in many dystrophies has been linked to the Ca^2+^-overload due to the SR calcium storage function alteration [83]. On the other hand, there is much evidence in the literature that mitochondria control collagen gene expression through two interdependent epigenetic and non-epigenetic mechanisms [81]. Both DNA and histone demethylases are 2-OGDO enzymes. 2-oxoglutarate itself is a key metabolite in the Krebs cycle [81]. As an example, in human fibroblasts, the *Col6A2* gene expression is reduced by CpG DNA methylation leading to an alteration of the extracellular matrix [84]. Conversely, damaged extracellular matrix induces the over-expression of DNMT3, one of the major proteins inducing DNA hypermethylation and finely tunes the regulation of gene expression in rat smooth muscle cell [85]. Hence, it is rather tempting to speculate that mitochondrial defects in *col6a1* ^*ama605003*^ line will create the condition of a cross-talk between *col6a* genes expression and mitochondrial health. Then, in muscular dystrophy, collagen mutation might be expected to induce the alteration of SR and mitochondria, which in turn will alter collagen expression thus amplifying in a vicious circle the initial mutation effect. Such a process could account for the striking, patchy phenotype observed in histological and TEM sections. Similarly, such process will amplify the mitochondrial alteration due to the ageing process [81] accounting at least partially for the late aggravation of the symptoms observed in many dystrophy diseases. Such pathway cross-talk could also be implicated in the age-related quality decrease of collagen, as it would trigger alterations of the matrix by activation of metalloproteinases [86].

## Conclusion

Our data show that the *col6a1* ^*ama605003*^ zebrafish line develops a mild and progressive form of collagen VI-related disease thus modeling perhaps best human BM. Adult zebrafish has become a widely used model organism for behavioral genetics [87] and for pharmacological studies due to the relatively low cost of maintenance as compared to mammals. Also, experiments on zebrafish embryos and fry allow the application of powerful statistical tests because of the simple production of high embryo numbers, which are easy to obtain and treat with a substance of interest by bathing. Data on long-term track record of BM patients remain scarce although the disease displays a great phenotypic variability [88]. Thus this first collagen VI mutant zebrafish line, modeling the condition in ageing animals might be used to study the pathological changes from as early as 2 dpf, to late 9 mpf, or even older fish. More generally, the *col6a1* ^*ama605003*^ line represents a suitable model for extensive drug tests to search for collagen VI-related disorder cures. We would gladly freely provide the *col6a1* ^*ama605003*^ line to any interested laboratory, please send your request to amagen@inaf.cnrs-gif.fr.

## S1 Materials and Methods

### Touch-evoked escape response

Dechorionated fish fry at 2 dpf were placed in the centre of a circle with a diameter of 10 mm and their behavior upon a gentle touch on the tail was recorded using a Zeiss axio zoom stereoscope at one frame per 70 ms. The necessary time for each fry to exit the circle was measured.

### Birefringence

Fish at 3 dpf or 4 dpf raised in embryo medium with or without methylcellulose were anesthetized with 0.5 mg/mL tricaine (MS222) buffered with sodium bicarbonate. They were immobilized in 1% low-melting agarose on microscope slides and placed between two polarizers (3D lens, Taiwan), then photographed with a Zeiss axio zoom stereoscope or a Nikon AZ 100 zoom microscope.

### Fluorescent phalloidin staining of F-actin

Zebrafish fries at 3 dpf were euthanized in a lethal concentration of tricaine, fixed in 4% PFA overnight at 4°C, permeabilized in 5% Triton X-100/PBS followed by F-actin staining using phalloidin conjugated to rhodamine (Sigma) over night. Stained fries were mounted in 50% glycerol and observed under a Zeiss LSM780 confocal microscope.

## Supporting Information

S1 Fig Origin of putative difference between mutations found in somatic and germline cells of zebrafish injected with TALENs. Upon injection of the TALEN-containing solution in fish embryos at the one-cell stage, different mutations are induced in some of the cells of the embryos (e. g. M1-M6) while no mutations arise in other cells (empty circles). The proportions of independently mutated and unmodified cells are variable and lead to the generation of mosaic fish. Lysis and genotyping of a sample of the injected embryos reveals the presence of some of the mutations in the F0 embryos. Similarly, lysis and genotyping of a fin clip of adult F0 fish detects some of the mutations present in somatic cells of these fish. These somatic mutations may be the same or different as compared to the mutations present in the germline cells of the fish. Thus, lysis and genotyping of a sample of the progeny, F1 embryos, may reveal the presence of identical or different mutations as compared to the mutations found in somatic cells of F0 parents. Also, mutations in heterozygous non-mosaic F1 fish raised to adulthood for the establishment of fish lines to analyse (*) may differ or not from the mutations found in F1 embryos. On the whole, genotyping at each stage of the process often detects only a subset of the mutations present in the pool of available ones and can reveal the sequence of some of the induced mutations. Other mutations may remain undetected and may appear at later stages of the process of establishment of a fish line.
(TIF)

S2 Fig Validation of somatic and heritable mutations in the col6a1 gene in zebrafish. (A) Number of mutations in the somatic cells of adult F0 fish and number of positive F1 fish found in a representative sample for each corresponding F0 fish. One of the mutations found in F1 embryos, M1 is overrepresented (ORM). Indicated are the numbers of F1 embryos containing the overrepresented mutation and the numbers of F1 embryos containing a different mutation. (B) Sequences of the five mutations found in the fin clips of F0 fish. The sequences of the overrepresented mutation (F1 3–2) and the other three targeted mutations found in F1 embryos are also shown. The corresponding numbers of F0 and F1 fish are indicated at the left and the number of deleted base pairs is indicated at the right.
(TIF)

S3 Fig The M1 mutation is absent out of the sequence of 10 wild type adult. The sequences of wild type adults, 5 females (top panel) and 5 males (bottom panel) are displayed together with their sequencing trace. The M1 over represented mutation, between vertical red lines (-2 bp, red box) is absent in these sequences. PCR primers used: (fwd) TCACTC CGTCTTCATTCAAAGATC, (rev) GTGATGGCAGCTTAAAGACG; sequencing primers (fwd) TCACTCCGTCTTCATTCAAAGATC.
(TIF)

S4 Fig No broad muscle disorganisation at 2 dpf in col6a1^ama60500^ mutants. Both wild type, HT and HM col6a1^ama605003^ fish showed bright birefringence indicative of high muscle organisation. This was confirmed with phalloidin-rhodamine staining revealing actin myofilaments.
(TIF)

S5 Fig Similar touch-evoked response between WT and HT and HM col6a1^ama605003^ mutants at 2 dpf. To assess locomotor activity of the fry, the touch-evoked response was measured in 2 dpf col6a1^ama605003^ mutant fry video-recorded at 70 ms/frame. The time between the touch and escape of the centre circle of 1 cm diameter was measured. There was no significant difference in touch-evoked response between the 3 genotypes.
(TIF)

S6 Fig Dying cells in col6a1^ama605003^ HM mutant at 5 mpf. TEM pictures of col6a1^ama605003^ HM mutant muscle showing (A) a dying myofibre with onset of cell membrane blebbing and disintegration. (B) Necrotic myofibre with ruptured plasma membrane and disorganized and degenerating organelles.
(TIF)

S7 Fig At 9 mpf, HM swam faster than WT and HT. Distribution histogram of cumulated time (s) WT and HT and HM col6a1^ama605003^ mutants swam at the indicated speed (S) in increment of 1 cm/s. This distribution was calculated from the analysis of video-recorded for 1 h free-moving conditioned fish. The 9 mpf HM swam at higher speed for longer time than WT and HT. For 3 mpf, N = 12, 16 and 11 and for 9 mpf N = 15, 19 and 7 for WT, HT and HM respectively.
(TIF)

S1 Supporting Information The supporting information comprises a supporting Materials and Methods (below) and 6 supporting Figures (see S1–S7 Figs Captions below). (DOCX)
