# Supplementary material for: A TALEN-Exon Skipping Design for a Bethlem Myopathy Model in Zebrafish
Source: PLoS One. 2015 Jul 29;10(7):e0133986. doi: 10.1371/journal.pone.0133986 (PMC4519248; doi:10.1371/journal.pone.0133986)
Supplement: S1 Supporting Information — (DOCX) [file pone.0133986.s008.docx]

**Supporting Information Captions**

**S1 Supporting Information.** The supporting information comprises a supporting Materials and methods (below) and 6 supporting Figures (see S1-S6 Figs. Captions below).

**S1 Materials and methods**

**Touch-evoked escape response**

Dechorionated fish fry at 2 dpf were placed in the centre of a circle with a diameter of 10 mm and their behavior upon a gentle touch on the tail was recorded using a Zeiss axio zoom stereoscope at one frame per 70 ms. The necessary time for each fry to exit the circle was measured.

**Birefringence**

Fish at 3 dpf or 4 dpf raised in embryo medium with or without methylcellulose were anesthetized with 0.5 mg/mL tricaine MS222 buffered with sodium bicarbonate. They were immobilized in 1% low-melting agarose on microscope slides and placed between two polarizers (3D lens, Taiwan), then photographed with a Zeiss axio zoom stereoscope or a Nikon AZ 100 zoom microscope.

**Fluorescent phalloidin staining of F-actin**

Zebrafish fries at 3 dpf were euthanized in a lethal concentration of tricaine, fixed in 4 % PFA overnight at 4°C, permeabilized in 5% Triton X-100/PBS followed by F-actin staining using phalloidin conjugated to rhodamine (Sigma) over night. Stained fries were mounted in 50% glycerol and observed under a Zeiss LSM780 confocal microscope.

**Supporting Figure Captions**

**S1 Fig. Origin of putative difference between mutations found in somatic and germline cells of zebrafish injected with TALENs^TM^.** Upon injection of the TALEN^TM^-containing solution in fish embryos at the one-cell stage, different mutations are induced in some of the cells of the embryos (e. g. M1-M6) while no mutations arise in other cells (empty circles). The proportions of independently mutated and unmodified cells are variable and lead to the generation of mosaic fish. Lysis and genotyping of a sample of the injected embryos reveals the presence of some of the mutations in the F0 embryos. Similarly, lysis and genotyping of a fin clip of adult F0 fish detects some of the mutations present in somatic cells of these fish. These somatic mutations may be the same or different as compared to the mutations present in the germline cells of the fish. Thus, lysis and genotyping of a sample of the progeny, F1 embryos, may reveal the presence of identical or different mutations as compared to the mutations found in somatic cells of F0 parents. Also, mutations in heterozygous non-mosaic F1 fish raised to adulthood for the establishment of fish lines to analyse (*) may differ or not from the mutations found in F1 embryos. On the whole, genotyping at each stage of the process often detects only a subset of the mutations present in the pool of available ones and can reveal the sequence of some of the induced mutations. Other mutations may remain undetected and may appear at later stages of the process of establishment of a fish line.

**S2 Fig. Validation of somatic and heritable mutations in the col6a1 gene in zebrafish.** (A) Number of mutations in the somatic cells of adult F0 fish and number of positive F1 fish found in a representative sample for each corresponding F0 fish. One of the mutations found in F1 embryos is overrepresented. Indicated are the numbers of F1 embryos containing the overrepresented mutation and the numbers of F1 embryos containing a different mutation. (B) Sequences of the five mutations found in the fin clips of F0 fish. The sequences of the overrepresented mutation (F1 3-2) and the other three targeted mutations found in F1 embryos are also shown. The corresponding numbers of F0 and F1 fish are indicated at the left and the number of deleted base pairs is indicated at the right.

**S3 Fig. No broad muscle disorganisation at 2 dpf in col6a1^ama60500^ mutants.** Both wild type, HT and HM col6a1^ama605003^ fish showed bright birefringence indicative of high muscle organisation. This was confirmed with phalloidin-rhodamine staining revealing actin myofilaments.

**S4 Fig. Similar touch-evoked response between WT and HT and HM col6a1^ama605003^ mutants at 2 dpf.** To assess locomotor activity of the fry, the touch-evoked response was measured in 2 dpf col6a1^ama605003^ mutant fry video-recorded at 70 ms/frame. The time between the touch and escape of the centre circle of 1 cm diameter was measured. There was no significant difference in touch-evoked response between the 3 genotypes.

**S5 Fig. Dying cells in col6a1^ama605003^ HM mutant at 5 mpf.** TEM pictures of col6a1^ama605003^ HM mutant muscle showing a (A) necrotic myofibre with ruptured plasma membrane and disorganised and degenerating organelles. (B) Dying myofibre with onset of cell membrane blebbing and disintegration.

**S6 Fig. At 9 mpf, HM swam faster than WT and HT.** Distribution histogram of cumulated time (s) WT and HT and HM col6a1^ama605003^ mutants swam at the indicated speed (S) in increment of 1 cm/s. This distribution was calculated from the analysis of video-recorded for 1 h free-moving conditioned fish. The 9 mpf HM swam at higher speed for longer time than WT and HT. For 3 mpf, N = 12, 16 and 11 and for 9 mpf N=15, 19 and 7 for WT, HT and HM respectively.
